# Cellular Allies Against Glioblastoma: Therapeutic Potential of Macrophages and Mesenchymal Stromal Cells

**DOI:** 10.3390/pharmaceutics18010124

**Published:** 2026-01-19

**Authors:** Bruno Agustín Cesca, Kali Pellicer San Martin, Luis Exequiel Ibarra

**Affiliations:** 1 Departamento de Biología Molecular, Facultad de Ciencias Exactas, Fisicoquímicas y Naturales, Universidad Nacional de Río Cuarto (UNRC), Rio Cuarto X5800BIA, Argentina; bcesca@exa.unrc.edu.ar (B.A.C.); pellicerkali@gmail.com (K.P.S.M.); 2 Instituto de Biotecnología Ambiental y Salud (INBIAS), Universidad Nacional de Río Cuarto (UNRC) y Consejo Nacional de Investigaciones Científicas y Técnicas (CONICET), Rio Cuarto X5800BIA, Argentina

**Keywords:** glioblastoma, macrophages, mesenchymal stromal cells, extracellular vesicles, tumor microenvironment, blood–brain barrier, cell-based delivery, biomimetic nanomedicine, immunomodulation, translational therapy

## Abstract

**Background/Objectives**: Glioblastoma (GBM) remains the most aggressive primary brain tumor in adults, with limited therapeutic options and poor prognosis despite maximal surgery, radiotherapy, and chemotherapy. The complex and immunosuppressive tumor microenvironment, pronounced intratumoral heterogeneity, and the presence of the blood–brain barrier (BBB) severely restrict the efficacy of conventional and emerging therapies. In this context, cell-based strategies leveraging macrophages, mesenchymal stromal cells (MSCs), and their derivatives have gained attention as “cellular allies” capable of modulating the GBM microenvironment and acting as targeted delivery platforms. **Methods**: This review systematically analyzes preclinical and early clinical literature on macrophage- and MSC-based therapeutic strategies in GBM, including engineered cells, extracellular vesicles (EVs), membrane-coated nanoparticles, and hybrid biomimetic systems. Studies were selected based on relevance to GBM biology, delivery across or bypass of the BBB, microenvironmental modulation, and translational potential. Evidence from in vitro models, orthotopic and syngeneic in vivo models, and available clinical trials was critically evaluated, with emphasis on efficacy endpoints, biodistribution, safety, and manufacturing considerations. **Results**: The reviewed evidence demonstrates that macrophages and MSCs can function as active therapeutic agents or delivery vehicles, enabling localized oncolysis, immune reprogramming, stromal and vascular remodeling, and enhanced delivery of viral, genetic, and nanotherapeutic payloads. EVs and membrane-based biomimetic platforms further extend these capabilities while reducing cellular risks. However, therapeutic efficacy is highly context-dependent, influenced by tumor heterogeneity, BBB integrity, delivery route, and microenvironmental dynamics. Clinical translation remains limited, with most approaches at preclinical or early-phase clinical stages. **Conclusions**: Cell-based and cell-derived platforms represent a promising but still evolving therapeutic paradigm for GBM. Their successful translation will require rigorous biomarker-driven patient selection, improved models that capture invasive GBM biology, scalable GMP-compliant manufacturing, and rational combination strategies to overcome adaptive resistance mechanisms.

## 1. Introduction

**Figure 1:**
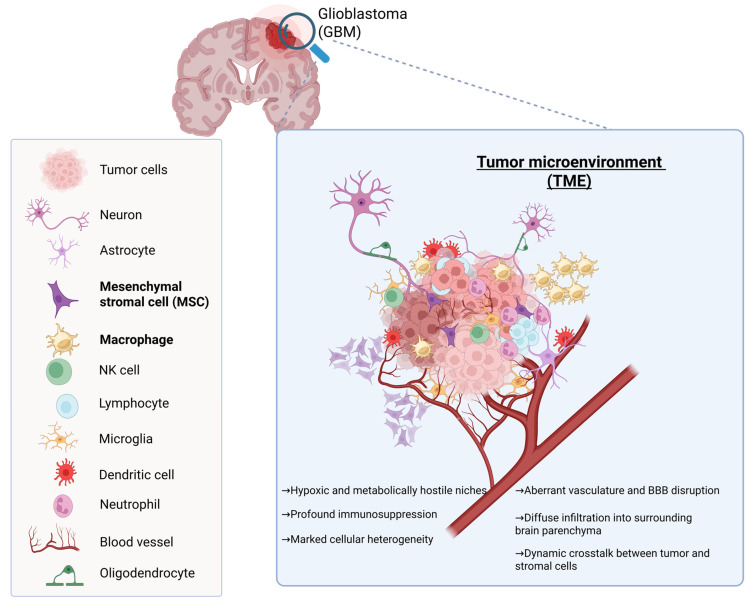
Cellular composition and defining features of the GBM TME. Schematic representation of the GBM microenvironment, highlighting the complex interplay between malignant tumor cells and diverse neural, stromal, vascular, and immune components. Diffuse infiltration of tumor cells into the surrounding brain parenchyma and sustained bidirectional crosstalk between tumor and stromal cells further contribute to therapeutic resistance and disease recurrence. Created in BioRender. Ibarra, L. (2026) https://BioRender.com/a6gw7p9 (accessed on 12 December 2025).

Glioblastoma (GBM) remains the most aggressive and lethal primary brain tumor in adults. Despite maximal surgical resection followed by radiotherapy and temozolomide (TMZ), median survival rarely exceeds 15–18 months, and long-term survival remains exceptional [1,2,3]. The dismal prognosis reflects the convergence of several biological hallmarks: extensive cellular and molecular heterogeneity, diffuse infiltration into eloquent brain structures, intrinsic and acquired therapeutic resistance, and a profoundly immunosuppressive tumor microenvironment (TME) (Figure 1) [4,5]. Current therapies fail to eradicate infiltrative tumor cells that migrate beyond the resection cavity, and most systemic agents are hindered by the restrictive properties of the blood–brain barrier (BBB) [6,7,8]. Consequently, GBM continues to pose an urgent clinical challenge, with limited therapeutic innovation over the last two decades and a critical need for approaches capable of overcoming immune evasion, drug-delivery barriers, and resistance pathways [9,10].

The TME comprises neurons, astrocytes, oligodendrocytes, microglia, mesenchymal stem/stromal cells (MSCs), macrophages, dendritic cells, neutrophils, natural killer (NK) cells, lymphocytes, and aberrant tumor-associated vasculature. These cellular elements collectively shape a highly dynamic and heterogeneous niche characterized by profound immunosuppression, hypoxic and metabolically hostile regions, extracellular matrix remodeling, and abnormal angiogenesis with partial BBB disruption.

Cell-based therapies have gained increasing attention as a means to address several of GBM’s most refractory features. Unlike conventional drugs, immune and stromal cells possess intrinsic abilities highly relevant to therapeutic design: tumor tropism, dynamic adaptation to inflammatory cues, sustained secretion of therapeutic molecules, and direct engagement with malignant and immune populations within the TME [11,12]. Macrophages and MSCs are of particular interest because they naturally infiltrate GBM, occupy key functional niches, and exert powerful immunomodulatory and paracrine effects. Their dual roles in promoting or restraining tumor progression underscore the importance of understanding and potentially reprogramming their biology. Advances in genetic engineering, synthetic biology, and biomaterial integration now allow these cells to serve as vectors for targeted drug delivery, cytokine release, local immune activation, and tumor-specific cytotoxicity. Together, these attributes position macrophages and MSCs as promising “cellular allies” capable of bypassing the BBB, modulating the TME, and delivering localized multimodal therapy.

This review synthesizes the current state of macrophage- and MSC-based therapeutic approaches for GBM by integrating evidence from molecular studies, in vitro models, in vivo systems, and emerging translational research. Literature was identified through a structured search of PubMed, Web of Science, and Scopus, focusing on studies published in the last decade, including recent articles published in 2025, and emphasizing mechanistic insights, engineering strategies, biodistribution, and therapeutic efficacy. Preference was given to peer-reviewed original research, high-impact reviews, and studies using clinically relevant models such as orthotopic xenografts, syngeneic systems, or advanced organoid platforms. Clinical trials involving macrophage or MSC platforms—including engineered variants, viral carriers, and combinatorial regimens—were also systematically examined. Publications without mechanistic detail, lacking reproducible methodology, or not directly involving macrophage or MSC biology in GBM were excluded. This curated approach allows a balanced, evidence-driven assessment of both opportunities and limitations of cell-based strategies.

The aim of this review is to critically evaluate the therapeutic potential of macrophages and MSCs in GBM, examining their biological roles, engineering strategies, translational challenges, and clinical progress. Following this introduction, Section 2 outlines the fundamental biology and heterogeneity of macrophages and MSCs. Section 3 analyzes their functional integration into the glioma microenvironment, including homing mechanisms, immunomodulation, vascular remodeling, and paracrine communication. Section 4 dissects the mechanistic basis of their therapeutic actions, while Section 5 surveys current and emerging cell-based strategies—unmodified, genetically engineered, and used as delivery vectors or combinatorial agents including the use of extracellular vesicles (EVs) or cell membranes for nanotechnology-based and biomimetic platforms that interact with or derive from these cells. Section 6 provides a critical assessment of current strategies designed to address the major biological constraints limiting cell-based therapies, including BBB permeability, immunosuppressive features of the TME, insufficient intratumoral penetration and retention, and the profound cellular and molecular heterogeneity of GBM. Section 7 evaluates preclinical models and translational metrics. Section 8 reviews the current clinical landscape of macrophage- and MSC-based therapies in GBM, highlighting early-phase trials that establish feasibility, safety, and proof-of-concept for engineered myeloid cells and MSC-mediated delivery, while underscoring that clinical translation remains in its infancy. Section 9 critically examines the translational gaps that currently limit clinical implementation, highlighting the lack of robust biomarkers, quantitative cell-tracking methodologies, and predictive stratification tools required to link biodistribution, immune modulation, and therapeutic efficacy in the context of highly heterogeneous GBM. Section 10 then addresses the biological safety risks and clinical challenges associated with cell-based and EV-based therapies, including immune-mediated toxicities, off-target effects, tumor-promoting liabilities, and neuroinflammatory complications unique to the intracranial setting. Finally, Section 11 discusses the manufacturing, scalability, and regulatory considerations necessary for the clinical translation of these advanced therapeutic platforms.

Together, these sections provide a comprehensive and critical framework for understanding how macrophages and MSCs can be harnessed, or reprogrammed, as therapeutic allies against GBM.

## 2. Biology of the Cellular Allies

### 2.1. Macrophage Phenotypes and Plasticity

Macrophages are key immune cells that reside in virtually all tissues, where they not only defend against pathogens but also contribute to tissue repair and the maintenance of homeostasis [13]. These cells originate from diverse hematopoietic progenitors, a feature that endows them with a broad range of functions and activation states that dynamically adapt to environmental cues and signaling inputs [14,15]. As part of the first line of defense, macrophages respond to a wide repertoire of endogenous and exogenous signals. Depending on the stimuli they detect, they can adopt distinct functional states or phenotypes, modulating both immune responses and local tissue balance. In the tumor context, both the microenvironment and infiltrating immune populations exert substantial influence on disease evolution. Consequently, macrophages assume a dualistic role, functioning either as immunosuppressive mediators or as facilitators of tumor progression, contingent upon the surrounding molecular signals [13,16].

For many years, macrophages were classified according to a binary M1/M2 model. M1 macrophages are associated with a proinflammatory profile characterized by the production of IL-12, TNF-α, and reactive oxygen species, whereas M2 macrophages exhibit an anti-inflammatory phenotype involved in tissue repair and the secretion of IL-10, TGF-β, and arginase-1 [17,18]. However, this dichotomous framework is now considered overly simplistic, as single-cell transcriptomic studies have revealed that macrophages display a far greater degree of functional diversity than the M1/M2 paradigm can adequately explain.

Tissue-resident macrophages exhibit distinct functions and gene-expression profiles compared with macrophages located in other organs. Moreover, macrophages positioned in different anatomical niches, or even within different microregions of the same tissue, display remarkable heterogeneity, particularly when comparing resident populations with those newly recruited from the circulation. This heterogeneity is strongly shaped by differential expression of lineage- and niche-specific markers, as revealed by single-cell transcriptomic and proteomic analyses, mass cytometry, and epigenetic profiling [19].

### 2.2. MSCs Identity, Sources and Heterogeneity

MSCs, also referred to as mesenchymal stem cells [20], are non-hematopoietic, multipotent cells derived from both adult and neonatal tissues [21,22]. In the first case, they reside in niches of adipose tissue, bone marrow, lungs, skeletal muscle, peripheral blood, periodontal ligament, heart, and gingiva. In the second case, MSCs are found in the umbilical cord, Wharton’s jelly, and placenta [20,21,22,23].

The main function of MSCs in adult tissues is to stimulate wound healing and reduce inflammation. However, antimicrobial, antifibrotic, and pro-regenerative properties have also been described, exerting effects on processes such as angiogenesis, proliferation, and immune modulation [24]. In addition, neonatal MSCs exhibit neuroprotective, fibroblast- and keratinocyte-stimulating, pro-angiogenic, and tissue-vascularizing activities during development, contributing to essential functions such as alveolar maturation and immune tolerance [25,26,27,28,29,30].

The multilineage potential of MSCs enables their differentiation into chondroblasts, osteoblasts, and adipocytes. This trilineage differentiation capacity has been demonstrated in vitro using lineage-specific induction media supplemented with (1) dexamethasone, β-glycerophosphate, and ascorbate for osteogenesis, (2) insulin and IBMX for adipogenesis, and (3) TGF-β3 for chondrogenesis [31,32]. This property is one of the formal criteria for defining MSCs according to the International Society for Cellular Therapy (ISCT) [33]. In vivo evidence of trilineage differentiation has also been reported, although it is highly dependent on the tissue microenvironment. For instance, human MSCs implanted in hydroxyapatite scaffolds can generate mature bone in immunodeficient mice [34]. Similarly, MSCs subjected to chondrogenic induction can preserve their cartilaginous phenotype and produce structurally stable hyaline cartilage in murine models [35,36]. Adipose-derived MSCs can also contribute to the reconstruction of functional adipose tissue when implanted in compatible niches [37,38]. Nevertheless, direct differentiation of MSCs both in vitro and in vivo is limited, as the induced phenotypes are often not sustained over time. Consequently, it is believed that the predominant role of MSCs is paracrine, mediated by the secretion of trophic factors rather than by extensive differentiation into terminal lineages.

Beyond classical trilineage differentiation, MSCs display remarkable plasticity. Reports have shown their ability to differentiate under specific culture conditions into hepatocytes [39,40], neuroblasts [41], neurons [42,43], endothelial cells [44], and cardiomyocytes [45]. Most of these differentiations occur under controlled conditions, and functional in vivo confirmation remains limited.

MSCs display adherence to plastic surfaces under standard in vitro culture conditions and are capable of forming fibroblast-like colonies. According to the ISCT, MSCs must express the surface markers CD105, CD73, and CD90, while lacking expression of hematopoietic markers such as CD45, CD34, CD14, CD11b, HLA-DR, CD79α, and CD19 [33]. In practice, however, this defining criterion is not fully consistent, as the phenotypic profile of MSCs is heterogeneous and largely influenced by the tissue source and microenvironmental context. For example, some studies have shown that the expression of CD105 varies among MSCs derived from different tissues [46]. Moreover, adipose-derived MSCs may initially express CD34 at early stages of isolation, which is progressively lost during subsequent in vitro passages [47]. Likewise, HLA-DR expression can be induced in umbilical cord MSCs under inflammatory stimulation [48]. In bone marrow–derived MSCs, CD271 is highly expressed in freshly isolated cells but decreases with successive passages [49]. In addition, CD146 expression is heterogeneous and delineates a distinct subpopulation of MSCs with demonstrated abilities for in vivo bone formation and trans-endothelial migration (TEM), underscoring its relevance for clinical strategies aimed at bone tissue regeneration [50].

In adipose-derived MSCs, in addition to the progressive loss of CD34, a strong expression of CD36 has been observed. This surface protein regulates lipid metabolism and inflammatory functions [51]. Conversely, placenta-derived MSCs express immunoregulatory molecules such as PD-L1 and PD-L2 [29,30], reflecting their physiological role in promoting immune tolerance during fetal development. This phenotypic variability translates into functional heterogeneity; thus, a given MSC population may contain distinct subpopulations depending on the donor, tissue of origin, and in vitro isolation or expansion conditions [20,52]. Moreover, single-cell transcriptomic analyses have revealed both inter- and intratissue heterogeneity among MSCs [23,52,53]. This documented heterogeneity poses significant challenges for the reproducibility and consistency of experimental findings, leading to ongoing debate regarding the translation of MSC-based approaches into clinically standardized therapies [53].

Moreover, it has been documented that MSCs undergo progressive cellular senescence during prolonged culture, accompanied by a gradual decline in their immunoregulatory capacity due to reduced expression of the immunosuppressive molecule PD-L1 [54].

Collectively, these observations indicate that MSCs are not inherently or universally immunosuppressive; rather, their effects are determined by their activation state, microenvironmental signals, and tissue of origin. This reinforces the functional variability reported across studies and underscores the importance of context in shaping MSC immunobiology.

## 3. Tumor-Associated Macrophages (TAMs) and MSCs Within the Glioma Microenvironment

### 3.1. Functional Diversity of TAMs in GBM Microenvironment

TAMs constitute a highly plastic and functionally diverse population that dynamically adapts to the TME. Depending on spatial localization, cytokine milieu, and metabolic conditions, TAMs can exhibit transcriptional programs ranging from proinflammatory and antitumor to immunosuppressive and pro-tumorigenic states [13,55]. In GBM, for instance, TAMs represent up to 30–50% of the total tumor mass, originating both from resident microglia and infiltrating bone marrow-derived macrophages (BMDMs) [56,57,58]. These subsets can coexist but differ in ontogeny, gene expression profiles, and immunological functions.

Until recently, studies on their activation states provided only a broad overview, relying primarily on bulk RNA analyses from individual tumor biopsies. With the advent of single-cell transcriptomics, it is now evident that GBM contain multiple TAM subpopulations that do not conform strictly to the classical M1/M2 polarization model [59]. Instead, these macrophages occupy intermediate activation states shaped by hypoxia, metabolic cues, and continuous crosstalk between tumor and stromal cells. This dynamic perspective portrays TAMs as a highly adaptable population capable of functional reprogramming in response to changes within the TME.

Furthermore, transcriptomic and phenotypic analyses have revealed clear distinctions between BMDMs and resident microglia, using markers such as *TMEM119* and *MHC-II* genes. Resident microglia-derived TAMs stably express genes associated with yolk sac embryonic lineage, including *TMEM119*, *P2RY12*, *SALL1*, *CX3CR1*, *TREM2*, and *GPR34*, and exhibit relatively low levels of CD45 and MHC-II [60,61]. In contrast, infiltrating BMDMs are characterized by high expression of CD45, CD49d (ITGA4), CCR2, CD14, CD163, and CD206 (MRC1), along with increased induction of MHC-II molecules such as HLA-DRA, HLA-DRB1, and HLA-DPA1 [62]. The relative proportion of these macrophage populations varies across GBM molecular subtypes. For instance, mixed tumors exhibit higher infiltration of BMDMs, whereas microglia predominate in the proneural and classical subtypes, being more activated or suppressed, respectively [59]. Further studies have delineated the dual ontogeny and extensive functional diversity of TAMs in GBM, identifying key signaling pathways such as CSF1R and STAT3 that govern their recruitment, survival, and polarization. These insights have prompted the development of therapeutic strategies aimed at targeting these pathways to shift the balance within the TME from a predominantly protumoral state toward one that favors antitumor immunity [63,64].

### 3.2. Phenotypic Adaptation of MSCs in GBM Microenvironment

MSCs have emerged as key stromal constituents of the glioma TME, where they exert profound effects on tumor biology, immune modulation, and microenvironmental remodeling. Far from being passive bystanders, glioma-associated MSCs (GA-MSCs) actively influence glioma progression through direct interactions with tumor cells [65], extensive paracrine signaling, and reciprocal crosstalk with immune cells, most notably macrophages [66]. Their presence and functional states within gliomas are strongly associated with enhanced malignancy, therapeutic resistance, and poor clinical outcomes [67].

MSCs isolated from glioma tissues display specialized phenotypes that support tumor growth and shape the architecture of the TME. GA-MSCs promote glioma cell proliferation, migration, and invasion by secreting growth factors, remodeling the extracellular matrix (ECM), and participating in the construction of the tumor vasculature [65,68]. Several studies have shown that MSCs can differentiate into pericyte-like cells, thereby stabilizing aberrant neovessels and facilitating tumor angiogenesis [69,70]. In parallel, MSCs contribute to ECM reorganization through deposition of collagen, fibronectin, and matrix metalloproteinases, enhancing tumor infiltration into surrounding brain tissue [71,72]. The heterogeneity of GA-MSCs further amplifies their functional impact. Subpopulations defined by markers such as CD90^high^ and CD90^low^ exhibit distinct biological behaviors, wherein some subsets preferentially drive tumor cell proliferation and migration, while others play a more prominent role in vascular support [73]. This phenotypic diversity highlights the adaptive potential of MSCs within the evolving glioma microenvironment.

MSCs exert many of their protumoral functions through paracrine secretion and EVs signaling. Cytokines such as IL-6 are abundantly released by GA-MSCs and reinforce tumor cell survival, invasion, and resistance to therapy through activation of STAT3, NF-κB, and other oncogenic pathways. In addition, MSC-derived exosomes transport tumor-modifying microRNAs, including miR-1587 and miR-191, which increase GSC self-renewal and tumorigenicity [74,75]. These EV-mediated interactions help maintain the stem-like reservoir of glioma cells, contributing to recurrence and therapeutic resistance.

The interactions between MSCs and TAMs represent one of the most influential immunoregulatory axes in gliomas [76]. Recently, it was demonstrated that the fusion of GA-MSCs with glioma cells enhances the secretion of CSF1, a key mediator of macrophage recruitment and M2-like polarization [77]. This process is regulated in part by m6A RNA modification and the activity of the demethylase obesity-associated protein, which collectively modulate CSF1 expression and amplify macrophage infiltration.

Macrophages, in turn, exert reciprocal effects on glioma and MSC behavior. Through the secretion of oncostatin M, TAMs induce mesenchymal-like transcriptional states in glioma cells via sustained activation of STAT3, thereby reinforcing tumor invasiveness and driving the emergence of more aggressive cellular phenotypes [78]. This bidirectional signaling establishes a reinforcing loop that strengthens the immunosuppressive and protumoral characteristics of the TME.

Further evidence indicates that macrophages enhance MSC chemotaxis toward tumor sites, while MSCs themselves modulate macrophage phenotype and function. Through the release of chemokines, cytokines, and EV-associated factors, MSCs skew macrophages toward an M2-like, immunosuppressive state, promoting immune evasion and supporting tumor maintenance [79]. This reciprocal chemotaxis and mutual reprogramming create a spatial and functional niche enriched in suppressive myeloid cells, aligning MSC and macrophage activities in favor of tumor progression.

The convergence of MSC-mediated microenvironment remodeling and macrophage polarization generates a powerful synergy that accelerates glioma progression. MSC-derived cues enhance TAM recruitment and suppress anti-tumor immunity, while macrophage-derived signals promote MSC infiltration and functional adaptation to the tumor milieu. This co-evolutionary interaction sustains an immunosuppressive environment characterized by elevated IL-10, CCL2, CSF1, and STAT3 pathway activation, collectively fostering angiogenesis, stemness, invasion, and resistance to therapy.

**Figure 2:**
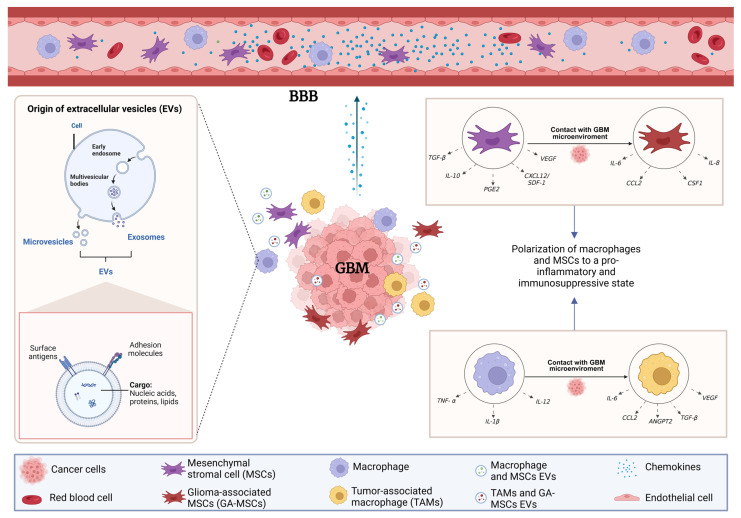
Reciprocal crosstalk between TAMs, MSCs, and EVs within the GBM microenvironment. The schematic illustrates the dynamic interactions between MSCs, macrophages, and glioma cells in the GBM tumor microenvironment. Both MSCs and macrophages undergo functional reprogramming upon exposure to tumor-derived cues, giving rise to glioma-associated MSCs (GA-MSCs) and tumor-associated macrophages (TAMs) with predominantly protumoral phenotypes. These cells secrete cytokines, chemokines, and extracellular vesicles (EVs) that mediate paracrine communication, modulate immune polarization, and reinforce angiogenesis, invasion, and immunosuppression. EVs originating from MSCs and macrophages carry bioactive cargo, including proteins, lipids, and nucleic acids, contributing to long-range signaling within the tumor niche. Together, these bidirectional interactions establish a self-sustaining stromal–immune network that supports GBM progression and therapeutic resistance. Created in BioRender. Ibarra, L. (2026) https://BioRender.com/wm2bf6h (accessed on 12 December 2025).

These reciprocal interactions between MSCs, macrophages, and glioma cells are further amplified by extracellular vesicle (EV)–mediated communication. As schematically illustrated in Figure 2, both MSCs and macrophages dynamically adapt their secretory and functional profiles upon contact with the GBM microenvironment, releasing EVs enriched in cytokines, chemokines, and regulatory nucleic acids that reinforce immunosuppression, angiogenesis, and tumor invasion. This bidirectional EV-driven signaling contributes to the co-evolution of stromal and immune compartments, consolidating a protumoral niche that supports GBM progression.

### 3.3. Tumor Tropism and Homing Mechanisms for Macrophages and MSCs

The GBM microenvironment exerts a powerful chemotactic influence on multiple stromal and immune cell populations, most prominently monocytes, neutrophils, TAMs, and MSCs [56]. Although these cell types differ in ontogeny and function, they share a remarkable capacity to sense inflammatory cues, migrate along chemokine gradients, and accumulate in highly specialized tumor niches. Understanding these homing mechanisms is central to deciphering GBM biology and to exploiting these cells as potential therapeutic allies.

Microglia-derived TAMs are enriched in proinflammatory gene programs, contribute to ECM remodeling, and are typically localized at the tumor periphery. These cells participate in antigen presentation and can partially activate T cells, thereby supporting tissue homeostasis and elements of immune surveillance [80,81,82]. In contrast, monocyte-derived TAMs predominate in necrotic and perivascular regions of the tumor, particularly in advanced or recurrent gliomas [83]. They exhibit pronounced immunosuppressive signatures, upregulate cytokines such as IL-10, and actively promote angiogenesis and tumor invasion [80,84]. Finally, their accumulation is associated with higher tumor grade and poorer clinical prognosis [83,85]. In addition to soluble chemokine gradients, EV-mediated signaling contributes to the spatial organization and functional conditioning of recruited stromal cells. As depicted in Figure 2, EVs derived from MSCs and TAMs participate in long-range communication within the tumor microenvironment, shaping macrophage polarization states and reinforcing chemotactic loops that promote sustained cellular recruitment.

As previously described, the GBM microenvironment is characterized by extensive accumulation of monocytes and macrophages [83]. This recruitment is orchestrated by multiple chemotactic signaling networks and physical mechanisms that support endothelial extravasation and migration from the circulation into tumor tissue [56].

Among the most thoroughly characterized axes, the CCL2–CCR2 and CSF1–CSF1R pathways act as principal drivers of circulating monocyte recruitment and their subsequent differentiation into TAMs. Preclinical studies have shown that gliomas secrete CCL2 (MCP-1) and other chemokines to attract CCR2^+^ monocytes from the bloodstream. Pharmacological disruption of this pathway reduces myeloid infiltration and prolongs survival in murine GBM models, highlighting its therapeutic potential [86]. Similarly, CSF1-dependent signaling regulates TAM survival and polarization, and pharmacological inhibition of CSF1R partially reprograms the immunosuppressive phenotype of TAMs, although compensatory mechanisms often limit sustained efficacy [63].

Beyond these canonical pathways, CXCL12–CXCR4 signaling also contributes to myeloid tropism, guiding TAM migration toward hypoxic and metabolically active regions. Within these niches, TAMs preferentially accumulate in perinecrotic and perivascular areas, where they promote angiogenesis and tissue remodeling. Their physical translocation from the bloodstream into the tumor parenchyma depends on biomechanical forces, integrin engagement, chemokine gradients, and adhesion molecules such as ICAM-1 and VCAM-1 [87,88,89]. TAMs further enhance endothelial cell proliferation and migration through secretion of proangiogenic factors, such as VEGFA, CXCL8 (IL-8), FGF2, PDGF, TGF-β, EGF, among others [90,91]. Hypoxic regions of GBM exacerbate these effects through overexpression of HIF-1α, which induces VEGFA and reinforces proangiogenic TAM activity [92].

These chemotactic programs operate within a structurally compromised vascular landscape, an intrinsic hallmark of GBM. BBB disruption acts synergistically with chemokine gradients, lowering the physical barriers to monocyte entry and thereby intensifying the dominance of the CCL2–CCR2 axis in regulating TAM accumulation. The translational relevance of this synergy was demonstrated in an orthotopic rat model by Cho et al. (2019), who showed that tumor irradiation or necrosis increased CCL2 expression, leading to enhanced recruitment of CCR2^+^ monocytes [93]. Pharmacological inhibition of CCL2 with mNOX-E36, alone or combined with bevacizumab, decreased TAM infiltration and improved antiangiogenic efficacy, highlighting the therapeutic value of suppressing CCL2-dependent macrophage recruitment.

Recent progress in live-cell imaging, in vitro assays, and microfluidic platforms have enabled real-time, high-throughput analysis of immune cell TEM. These approaches frequently employ fluorescent THP-1 monocytes, monolayers of human umbilical vein endothelial cells (HUVECs), and three-dimensional endothelial-on-chip models, which together provide a versatile toolkit for examining the dynamics, adhesion, and migratory behavior of immune cells under both static and flow conditions [94,95]. Such models are particularly valuable for elucidating how different activation states influence cellular functions during TEM, as well as for dissecting the roles of cytokines and chemokines in regulating these processes.

More specifically, in the context of platforms developed for GBM research, DePalma et al. (2025) introduced a three-dimensional microfluidic model of the BBB composed of human endothelial cells and a tissue-mimetic hydrogel [96]. This system demonstrated that the presence of GBM cells alters BBB permeability and enhances immune cell adhesion to the endothelial surface [96]. Such findings indicate that this platform may be particularly valuable for investigating the dynamic interactions between monocytes/macrophages and the endothelium in a tumor-specific context. Similarly, Straehla et al. (2022) employed a vascularized GBM-on-a-chip microdevice incorporating endothelial cells, pericytes, astrocytes, and tumor spheroids [97]. This platform enabled detailed evaluation of nanoparticle (NP) trafficking and suggested that comparable models could be adapted to monitor monocyte migration toward tumor-associated vasculature [97]. Additional organ-on-a-chip systems featuring perfused endothelial barriers have already been used to examine human monocyte (THP-1) migration under physiologically relevant flow conditions [98]. These platforms allow systematic analysis of chemokine gradients, hemodynamic forces, and mechanisms underlying TEM.

MSCs also display a strong intrinsic tropism toward inflammatory sites and solid tumors, including GBM. Their recruitment is primarily governed by the SDF-1/CXCL12–CXCR4 axis, integrin-mediated adhesion (e.g., VLA-4), and matrix remodeling via MMP-2 and MMP-9 [99,100,101,102]. In the context of glioma, tumor-derived chemokines and cytokines establish robust chemotactic gradients that attract MSCs from peripheral or local stromal reservoirs. Both in vitro spheroid assays and in vivo orthotopic mouse models have demonstrated that MSCs migrate directionally toward GBM-secreted CXCL12 and accumulate in hypoxic or peritumoral zones characterized by elevated SDF-1 and TGF-β levels [103,104]. These homing properties position MSCs as both modulators of the TME and potential therapeutic delivery vehicle.

Taken together, macrophage and MSC recruitment to GBM reflects the convergence of specific chemotactic signals and structural vascular abnormalities that together generate highly permissive routes for stromal and immune cell infiltration. TAMs and MSCs respond to many of the same chemoattractants and environmental cues, albeit with distinct functional consequences once within the tumor. From a therapeutic standpoint, this duality can be exploited in two complementary ways: by inhibiting chemotactic pathways to reduce protumoral TAM burden, or by harnessing natural tropism to deliver engineered macrophages or MSCs loaded with antitumor agents deep into the tumor mass.

### 3.4. Immunomodulation and Reprogramming of the TME by TAMs and MSCs

#### 3.4.1. Reprogramming of Tumor-Associated Macrophages in TME

The immunological landscape of GBM is shaped by a complex interplay between tumor-derived signals, infiltrating immune cells, and stromal components such as TAMs and MSCs. Both cell types exhibit substantial plasticity and can be reprogrammed by the TME, acquiring either antitumor or, more frequently, immunosuppressive phenotypes that support tumor progression. Understanding how TAMs and MSCs are modulated within the TME is essential for the development of strategies aimed at restoring antitumor immunity in GBM.

Recent studies have demonstrated a bidirectional crosstalk between TAMs and glioma stem cells (GSCs) that drives tumor invasion, neovascularization, and chemoresistance [105]. TAMs secrete soluble mediators such as IL-6, TGF-β, and CCL2, which help maintain the stem-like phenotype of GSCs [106]. Conversely, GSCs produce signals that reinforce the immunosuppressive and protumoral phenotype of TAMs [107,108,109]. This reciprocal communication establishes a vicious cycle that promotes GBM progression, therapeutic resistance, and recurrence.

Among the central regulators of macrophage survival and polarization in GBM, the CSF1–CSF1R axis is one of the most extensively studied. Yan et al. (2017) demonstrated that pharmacological inhibition of CSF1R with PLX3397 markedly reduces TAM density and prolongs survival in murine glioma models [110]. However, some tumors developed adaptive resistance characterized by increased secretion of IGF-1 and compensatory activation of the PI3K pathway, ultimately limiting the durability of the therapeutic response. More recent work has highlighted that the response to CSF1R inhibition is influenced by the genetic drivers of the glioma, including alterations in *EGFR*, *NF1*, *PDGFRA*, and *IDH1*. These oncogenic programs not only dictate tumor growth but also shape the immune microenvironment by modulating the behavior of macrophages and microglia. In an important study, Rao et al. (2022) demonstrated that different molecular subtypes of glioma exhibit distinct TAM functional states and respond heterogeneously to CSF1R inhibition [111]. These findings suggest that the therapeutic efficacy of CSF1R-targeted strategies is highly context-dependent, and that molecular stratification may be required to identify patients who are most likely to benefit [111].

Complementary results were reported by Fermi et al. (2023) using patient-derived organoids [112]. Treatment with the CSF1R inhibitor GW2580 reduced expression of immunosuppressive genes such as *IL10* and *IL6*, while simultaneously activating IFN-γ– and NF-κB–associated pathways, collectively shifting TAM function toward an antitumor phenotype. TAMs exposed to CSF1R blockade also displayed enhanced phagocytic capacity and antigen-presentation potential, indicating effective immune reprogramming within the TME [112]. Together, these studies establish CSF1R inhibition as one of the most promising strategies to pharmacologically reprogram TAMs in GBM. However, resistance mechanisms and the transient nature of TAM repolarization remain significant challenges to durable therapeutic benefit.

#### 3.4.2. MSC Plasticity and Immune Reprogramming Within the Glioma Microenvironment

MSCs possess a robust immunoregulatory capacity that enables them to either suppress or activate distinct immune cell populations depending on the inflammatory milieu. Similar to macrophages, MSCs exhibit functional polarization, a phenomenon demonstrated both in vitro and in vivo. Activation of TLR4 in MSCs induces a proinflammatory phenotype known as MSC1. The MSC1 secretome promotes early neutrophil infiltration through the release of IL-6 and IL-8, followed by the recruitment of macrophages and fibroblasts that facilitate tissue repair [113]. In contrast, stimulation of TLR3, localized within endosomes, the endoplasmic reticulum (ER), and on the cell membrane, drives MSCs toward an anti-inflammatory phenotype (MSC2). MSC2s modulate T cells and reprogram macrophages toward an anti-inflammatory state through soluble mediators as well as direct cell–cell interactions mediated by ICAM-1 and galectin-3 [114]. MSCs also suppress T-cell receptor (TCR) signaling and inhibit the transcription of proinflammatory cytokines in activated T cells through contact-dependent mechanisms involving ICAM-1/CD43 interactions [115]. Multiple studies have further shown that exposure to MSCs promotes the conversion of T cells toward a Treg-like phenotype [115,116].

In addition, the MSC secretome drives macrophage polarization toward an anti-inflammatory M2 phenotype, as previously described. In both cellular and animal models, this effect has been attributed to exosomal miRNAs, such as miR-21-5p [117], and activation of the IL-10/STAT3 pathway [118]. Importantly, MSC immunomodulatory activity is not uniform across all sources. Liver-derived MSCs, for example, exhibit a stronger ability to inhibit NK cell function than adipose- or bone-marrow-derived MSCs [119], reflecting source-dependent differences in their secretome composition. Although MSC immunosuppressive effects can be reproduced in vitro under standardized conditions, the magnitude of these effects varies depending on the tissue of origin and experimental parameters [120].

The interaction between MSCs and the GBM immune microenvironment is equally complex and remains one of the most debated aspects of stromal biology in gliomas as we previously described. Whereas naïve or uneducated MSCs may retain certain immunoregulatory or even antitumor properties, sustained exposure to the GBM milieu frequently reprograms them toward protumoral and immunosuppressive phenotypes. Evidence for this reprogramming was demonstrated by Pietrobono et al. (2024), who showed that tumor-conditioned environments alter the immunomodulatory behavior of MSCs by suppressing their ability to generate an adenosine-mediated immunosuppressive milieu [121]. Changes in PD-L1 expression have also been reported. Gao et al. (2023) observed that extended passaging of MSCs leads to progressive loss of immunoregulatory capacity associated with decreased PD-L1 expression [54]. Interestingly, this loss appears reversible and MSCs proliferating in the presence of GBM-derived signals regain PD-L1 expression and immunomodulatory potential [68]. These findings indicate that GBM-associated MSCs acquire a phenotype already shaped by cancer cells and the TME, reflecting the high degree of immune plasticity inherent to MSCs.

Despite these insights, studies specifically examining the secretome of GBM-associated MSCs remain limited. Most work relies on naïve or exogenous MSCs exposed to tumor-like conditions rather than MSCs isolated directly from GBM tissue. This gap is particularly relevant, given that TME-educated MSCs display functional and transcriptional adaptations that likely influence their paracrine impact on GBM progression.

Several studies have shown that MSCs educated by GBM can actively recruit immune cells and polarize them toward inflammatory or immunosuppressive states, with CD73 emerging as a central mediator of these effects [79,122,123]. Additionally, GBM cells can transfer microvesicles to MSCs, reducing the expression of antitumor microRNAs such as miR-100-5p, miR-9-5p, and let-7-5p. This exchange drives MSCs toward a cancer-associated fibroblast–like phenotype capable of secreting exosomes enriched in protumoral microRNAs [124,125].

On the other hand, a striking example of MSC–macrophage cooperation was reported by Liu et al. (2025), who showed that hybrid cells formed through fusion between GBM-associated MSCs and tumor cells recruit macrophages and polarize them toward an M2 phenotype via CSF1 secretion, thereby strengthening immunosuppression within the tumor niche [77].

Taken together, the evidence indicates that MSC immunoregulatory behavior is highly time- and context-dependent. Naïve MSCs may exert modest antitumor or immunoregulatory effects, but prolonged exposure to the GBM microenvironment drives phenotypic reprogramming that reinforces immunosuppression, TAM recruitment, and tumor progression. In parallel, TAMs themselves can be pharmacologically reprogrammed toward antitumor phenotypes through CSF1R inhibition, though resistance mechanisms may limit long-term benefit. The convergence of these findings underscores the dynamic nature of immune-stromal interactions in GBM and highlights the need for therapeutic strategies that simultaneously target both macrophage and MSC plasticity to effectively reshape the TME.

### 3.5. Vascular and Stromal Remodeling Effects

Vascular and stromal remodeling constitute hallmarks of GBM progression, fundamentally shaping tumor invasion, metabolic adaptation, and therapeutic resistance. Within this highly dynamic microenvironment, both TAMs and MSCs emerge as key stromal regulators that remodel the ECM, modify stromal stiffness, and orchestrate pathological angiogenesis. Although these roles are primarily protumoral, they also provide actionable therapeutic entry points for microenvironment-targeted interventions.

#### 3.5.1. TAM-Mediated Vascular and Stromal Remodeling

The invasive behavior of GBM is not solely dictated by tumor cell–intrinsic programs but is critically shaped by reciprocal interactions with stromal and immune components of the TME. Among these, TAMs emerge as dominant regulators of ECM remodeling, vascular reprogramming, and phenotypic plasticity, thereby creating permissive conditions for tumor cell migration, survival, and therapeutic resistance. Through the coordinated secretion of proteases, growth factors, cytokines, and matricellular proteins, TAMs actively reshape both the biochemical and biomechanical landscape of the GBM niche.

A convergent theme emerging from experimental studies is that TAM-derived signals engage central oncogenic pathways in glioma cells to directly enhance invasive behavior. As summarized in Table 1, macrophage-secreted epidermal growth factor (EGF) and EGFR ligands such as amphiregulin activate EGFR signaling in GBM cells, triggering downstream PI3K/Akt and ERK1/2 cascades that promote matrix metalloproteinase expression, ECM degradation, and perivascular dissemination. This bidirectional signaling circuit is reinforced by CSF-1/CSF-1R–dependent macrophage recruitment and by EGFR-driven induction of adhesion molecules, enabling coordinated macrophage–tumor cell migration within stromal and perivascular niches.

Beyond soluble growth factors, TAMs—particularly those polarized toward an M2-like phenotype—contribute to stromal remodeling through the secretion of matricellular proteins and EVs. These factors not only facilitate physical matrix remodeling but also induce transcriptional reprogramming of glioma stem-like cells toward mesenchymal, invasive, and therapy-resistant states. Hypoxic conditions further potentiate these effects by amplifying proangiogenic signaling [126] and EV-mediated transfer of regulatory RNAs that activate epithelial–mesenchymal transition programs, linking immune signaling to vascular and structural plasticity within GBM (Table 1).

Collectively, these mechanisms position TAMs as active catalysts rather than passive responders in GBM invasion, integrating ECM degradation, vascular remodeling, and tumor-intrinsic signaling into a unified invasive program. From a therapeutic perspective, this central role has motivated strategies aimed at disrupting TAM-mediated stromal remodeling. Inhibition of CSF1R signaling reduces macrophage density and attenuates proangiogenic and matrix-remodeling functions, resulting in partial vascular normalization in preclinical models [127,128]. However, recent evidence indicates that prolonged TAM depletion can elicit compensatory fibrotic remodeling that favors tumor recurrence, underscoring the context-dependent and temporally limited benefits of TAM-targeted monotherapies [129].

In line with these findings, TAMs are dominant drivers of stromal and vascular remodeling in GBM, particularly within hypoxic and perivascular tumor niches. They secrete a broad repertoire of proangiogenic mediators, including VEGF, FGF2, PDGF, IL-8, and TGF-β, as well as matrix metalloproteinases, which collectively promote endothelial proliferation, aberrant neovascularization, and ECM breakdown [130,131,132]. Hypoxia further amplifies these processes by stabilizing HIF-1α, inducing VEGF expression, and reinforcing TAM-mediated angiogenic programs [126].

In summary, TAM-driven vascular and stromal remodeling constitutes a core enabling axis of GBM invasion and progression. The pathways compiled in Table 1 provide a mechanistic framework for understanding how macrophage-derived signals orchestrate malignant dissemination and highlight actionable nodes that may be most effectively targeted through rational combination strategies designed to disrupt the invasive ecosystem of GBM.

**Table 1:** TAM-mediated vascular and stromal remodeling in GBM. Summary of key molecular mediators secreted or regulated by TAMs that drive ECM remodeling, angiogenesis, and invasive behavior in GBM models. The table highlights mechanisms, biological effects, experimental systems, and representative references.

TAM-Derived Factor	Mechanism/Pathway	Biological Effect in GBM	Experimental Model	Reference
TGFBI (BIGH3)	ECM interaction; integrin signaling	Enhances glioma cell invasion and motility	3D GBM co-culture model	[133]
VEGF, FGF2, PDGF, IL-8	Angiogenic signaling; HIF-1α- and HIF-2α driven expression	Aberrant neovascularization and vascular remodeling	Orthotopic GBM mouse models	[134,135,136]
EGF	EGFR activation; PI3K/Akt and ERK1/2 pathways	Upregulation of MMP-9, enhanced invasion and migration	In vitro and in vivo GBM models	[137]
MMP-9	ECM degradation downstream of EGFR signaling	Facilitates perivascular invasion	Glioma cell–macrophage co-cultures	[138]
M2-TAM EVs	Induction of EMT programs under hypoxia	Increased motility and mesenchymal transition	Hypoxic GBM models	[139]

Footnotes: TAMs, tumor-associated macrophages; ECM, extracellular matrix; GBM, glioblastoma; EMT, epithelial–mesenchymal transition. References are representative and correspond to studies discussed in the main text.

#### 3.5.2. MSC-Driven Stromal Remodeling and Vascular Modulation

MSCs also contribute substantially to ECM remodeling, stromal stiffening, and vascular dynamics in GBM, particularly after undergoing phenotypic reprogramming within the TME. These MSC-derived changes support tumor infiltration, therapeutic resistance, and microenvironmental restructuring. GBM-educated MSCs can upregulate LOX and COL1A1 through reprogramming of the CD40L/CD40 axis, resulting in a stiffer, collagen-rich stroma that enhances cellular infiltration [65]. Additionally, MSCs exposed to tumor-derived factors frequently acquire cancer-associated fibroblast (CAF)-like properties, including enhanced secretion of proangiogenic molecules and ECM components that promote vascular remodeling and support tumor expansion [124]. Metabolic crosstalk represents another mechanism by which MSCs remodel the GBM microenvironment. Nakhle et al. (2023) demonstrated that MSCs transfer mitochondria to GBM cells via tunneling nanotubes, increasing oxidative phosphorylation and ATP levels in tumor cells and promoting proliferation and TMZ resistance under metabolic stress [140]. Consistent with this concept of metabolically driven stromal support, Zhang et al. recently demonstrated that exosomal miR-21-5p derived from GA-MSCs directly suppresses PDHA1 expression in GBM cells, thereby enhancing glycolysis and further promoting GBM proliferation, migration, and invasion [141].

Furthermore, emerging evidence indicates that MSC–GBM hybrid cells can form within the TME, contributing to stromal remodeling and tumor progression through altered gene expression programs and enhanced secretory activity [77,142]. These observations highlight the extent to which MSCs become functionally rewired by sustained exposure to the tumor milieu, ultimately transforming into stromal components that actively sustain GBM aggressiveness.

### 3.6. Paracrine Signaling: Cytokines, Chemokines, and Growth Factors

Paracrine signaling is a central mechanism through which both TAMs and MSCs influence the GBM microenvironment. Through the release of cytokines, chemokines, growth factors, and EVs, these cell populations orchestrate a complex network of molecular interactions that regulate angiogenesis, immune suppression, metabolic adaptation, ECM remodeling, and tumor invasiveness. While many of these paracrine interactions reinforce tumor progression, others can exert context-dependent antitumor effects, reflecting the functional plasticity of stromal and immune cells in GBM. Although TAMs and MSCs arise from distinct lineages and exhibit different functional roles, their paracrine programs converge on shared pathways that reinforce GBM progression. Their mutual reinforcement is further evidenced by mechanisms in which MSC-derived cues enhance macrophage recruitment and polarization, while TAM-derived factors shape the phenotypic and secretory programs of MSCs.

This intertwined paracrine signaling establishes a self-sustaining loop that amplifies malignancy and creates a spatially organized, immunosuppressive and proinvasive niche.

#### 3.6.1. Paracrine Activity of MSCs with Dual Roles in Tumor Promotion and Suppression

The MSC secretome is composed of soluble factors, cytokines, chemokines, fibroblast and endothelial growth factors, as well as EVs. Collectively, these signals have been implicated in immunoregulation, angiogenesis, ECM remodeling, and modulation of oxidative stress pathways [143]. Among soluble mediators, TGF-β1 secreted by MSCs derived from bone marrow, adipose tissue, and umbilical cord has been shown to promote tumor proliferation in 3D models [144]. However, as discussed earlier, MSCs can also release antiproliferative or proapoptotic mediators depending on cellular context [145], highlighting their dual potential.

EV-mediated signaling also displays functional heterogeneity. Qui et al. (2023) found that GBM-associated MSCs secrete EVs enriched in miR-2, which increases CD73 expression in myeloid-derived suppressor cells and promotes immunosuppression and tumor progression through the PTEN/PI3K/AKT/HIF-1α axis in vitro and in vivo [79]. Likewise, Lv et al. (2025) reported enrichment of miR-191-5p in MSC-derived EVs that enhanced proliferation, migration, and invasiveness of GBM cells [74]. Complementary mechanisms have been proposed, such as paracrine activation of the B1R receptor by MSC-derived kinins, which further promote tumor cell migration and proliferation [146].

Nevertheless, paracrine activity is not uniformly protumoral. Jafari et al. (2023) found that adipose-derived MSC conditioned medium induced overexpression of oncogenes such as *SOX4* and *H19* in U87 cells, but paradoxically increased apoptosis [147]. Conversely, another study using adipose-derived MSC conditioned medium in HROG36, U87MG, and T98G cells observed reduced expression of invasion- and angiogenesis-related genes and decreased tumor cell invasiveness in a GBM model in chicken embryo chorioallantoic membrane [147]. These conflicting results highlight that the biological impact of MSC-derived signals depends on tissue origin, GBM subtype, and experimental context.

Overall, paracrine signaling by MSCs follows a recurrent pattern: functional outcomes are strongly influenced by MSC origin, degree of tumor education, and model system. This variability has enabled the identification of mechanistic drivers underlying both antitumor and protumoral effects, while motivating efforts to genetically engineer MSCs or their EV cargo to bias their activity toward tumor suppression and therapeutic drug delivery.

#### 3.6.2. Paracrine Activity of TAMs: Immune Suppression, Recruitment, and Oncogenic Reinforcement

Paracrine communication from TAMs is equally central to GBM biology and represents a major therapeutic target. M2-polarized TAMs secrete cytokines that reinforce immunosuppression and aberrant tissue repair, including IL-10 and TGF-β. TGF-β not only suppresses T and NK cell activity, but is a key promoter of GSC phenotype and EMT, enhancing invasion [148]. Similarly, CCL2 (MCP-1), produced by macrophages and tumor cells, acts as both a chemotactic signal for new monocytes and an autocrine factor sustaining protumoral TAM functions [149], supporting persistent tumor-promoting myeloid infiltration.

An illustrative example of the potency of paracrine signaling is provided by Liu et al. (2025), who demonstrated that GA-MSCs can fuse with tumor cells to generate hybrid cells that secrete elevated levels of CSF1 [77]. This increase, driven by m6A-modulated stabilization of CSF1 mRNA, promotes monocyte recruitment and M2 polarization. These findings emphasize that the GBM microenvironment is not a passive byproduct of tumor growth, but an active, evolving ecosystem in which malignant cells co-opt stromal components to sustain immunosuppression, invasion, and tumor progression.

Targeting these paracrine networks has shown therapeutic potential. Inhibition of the CSF1–CSF1R axis reduces macrophage recruitment and decreases secretion of protumoral cytokines such as IL-10 and IL-6, while enhancing IFN-γ and NF-κB pathway activation and promoting macrophage repolarization toward antitumor phenotypes [150]. Blockade of CCL2/CCR2, alone or in combination with immunotherapy, is also being actively explored to dismantle the immunosuppressive niche and restore T cell effector function.

As a result, therapeutic strategies that selectively disrupt these signaling axes, individually or combinatorially, represent promising avenues for reprogramming the GBM microenvironment.

## 4. Mechanisms of Therapeutic Action of TAMs and MSCs

The profound functional plasticity of TAMs and MSCs within the glioma microenvironment provides a mechanistic foundation for exploiting these cells as therapeutic targets or delivery platforms. As outlined in Section 3, both cell types undergo extensive transcriptional and phenotypic reprogramming in response to tumor-derived cues, enabling them to either support glioma progression or, under specific conditions, contribute to antitumor immunity and tumor suppression. These context-dependent behaviors arise from diverse mechanisms, including direct cytotoxic effector functions, metabolic and apoptotic reprogramming of glioma cells, immunomodulatory activity, and remodeling of the vascular and stromal compartments. Understanding these mechanisms is essential for designing rational therapeutic interventions capable of enhancing beneficial properties while mitigating protumoral activities. In the following subsections, we examine the principal therapeutic modes through which TAMs and MSCs can be harnessed or re-engineered to counteract GBM, focusing on direct antitumor actions, paracrine and metabolic modulation, immune reprogramming, and microenvironmental restructuring.

GBM exhibits pronounced molecular and histological heterogeneity that critically shapes the composition and functional state of stromal elements such as TAMs and GA-MSCs. Distinct molecular subgroups—classically described as proneural, classical and mesenchymal in transcriptional classifications—present differing tumor-intrinsic drivers (for example, PDGFRA/IDH alterations in proneural, EGFR amplification in classical, and NF1 loss/mesenchymal programs in the mesenchymal subtype) that correlate with vascular architecture, necrosis, hypoxia, and immune infiltration (Table 2). Importantly, the mesenchymal transcriptional state is frequently associated with elevated myeloid signatures, increased infiltration of bone-marrow-derived macrophages, and an immunosuppressive cytokine milieu (high CCL2/CSF1/IL-10), whereas proneural tumors often show comparatively lower myeloid burdens and distinct microglia-like signatures. These subtype-specific microenvironments have direct implications for cell therapy: tumors with high myeloid infiltration and permissive vasculature may be more accessible to macrophage- or MSC-based delivery strategies but may also present a stronger immunosuppressive barrier that requires concurrent myeloid reprogramming; conversely, tumors with intact BBB regions or low myeloid density may require ligand-guided carriers, intraparenchymal/intracavitary delivery, or strategies to transiently increase tumor permeability. Thus, translational development of macrophage- and MSC-based therapeutics should incorporate molecular (e.g., IDH, EGFR, NF1), epigenetic and immune readouts (TAM density, BMDM–microglia ratio, CSF1/CCL2 expression, PD-L1) together with radiological features (contrast enhancement, perfusion) to define stratification criteria and optimize patient selection.

### 4.1. Direct Anti-Tumor Activities

#### 4.1.1. Macrophage-Mediated Cytotoxicity, Phagocytosis, and Bystander Effects

Although TAMs are often associated with pro-tumoral functions, numerous studies have demonstrated that, under appropriate stimuli, they can also exert potent antitumor activity. Classically activated M1-like macrophages, typically induced by IFN-γ or lipopolysaccharide (LPS), display strong cytotoxic properties mediated by the production of nitric oxide (NO), reactive oxygen species (ROS), and proinflammatory cytokines such as TNF-α and IL-12. These mediators inflict oxidative damage on tumor cells while simultaneously enhancing adaptive immune responses by increasing antigen presentation and stimulating cytotoxic T lymphocytes [56].

In GBM models, activated macrophages have been shown to phagocytose tumor cells and secrete signals that amplify local immune responses. A promising therapeutic strategy to enhance these functions is the blockade of the CD47–SIRPα axis, a key “don’t eat me” signal that protects GBM cells from phagocytic elimination. Inhibition of CD47 restores macrophage phagocytic capacity and promotes tumor clearance in vivo [159]. However, von Roemeling et al. (2020) demonstrated that CD47 blockade alone produces only modest improvements with approximately a 10–20% increase in phagocytosis by BMDMs, indicating that CD47 inhibition must be complemented by additional interventions targeting tumor-cell intrinsic vulnerabilities [160].

A more effective strategy involves combining CD47 blockade with TMZ, the standard chemotherapeutic agent for GBM. TMZ induces ER stress in tumor cells, making them more susceptible to phagocytosis. This stress response is marked by elevated BiP, phosphorylated eIF2α, and CHOP, as well as the translocation of calreticulin to the cell surface, a hallmark “eat me” signal. ER stress induced by TMZ promotes immunogenic cell death (ICD) or at least the exposure of danger-associated molecular patterns (DAMPs), increasing macrophage-mediated phagocytosis and enhancing antigen presentation through activation of the cGAS–STING pathway in antigen-presenting cells. Through these mechanisms, the TMZ + anti-CD47 combination not only strengthens innate tumor clearance but also initiates more robust adaptive immune activation [160]. The enhanced phagocytosis observed with this combination triggers increased production of IL-1β and IFN-β and drives the expansion of tumor-specific T cells. However, this intensified immune activity can also promote adaptive resistance pathways in the TME. Sequential administration of anti-PD-1 has been shown to overcome this resistance, producing a more durable and potent antitumor response [160].

Together, these findings demonstrate that effective enhancement of macrophage antitumor activity in GBM requires restoring phagocytic engagement by neutralizing “don’t eat me” signals and simultaneously inducing tumor cell stress to expose “eat me” signals or trigger ICD. This dual approach integrates innate and adaptive immune responses, providing a strong conceptual framework for the development of macrophage-based therapies in GBM.

#### 4.1.2. MSC-Mediated Antitumor Activities

Despite the frequent association of MSCs with immunosuppressive and protumoral functions in the GBM microenvironment, a substantial body of evidence demonstrates that MSCs can also exert context-dependent antitumor activities. These effects arise from both direct interactions with glioma cells and a diverse array of paracrine mechanisms capable of modulating apoptosis, metabolism, autophagy, and invasion. Their homing capacity is not solely a physical or migratory phenomenon. Once MSCs enter the tumor mass, they may undergo phenotypic polarization, adopting immunosuppressive, pro-invasive, or antitumor functional states depending on the cues they encounter. Recent literature emphasizes that the role of MSCs in GBM is highly context-dependent, varying according to their tissue of origin, mode of interaction, and duration of exposure to the TME. For that reason, the dual nature of MSC behavior underscores their functional plasticity and highlights the need to examine their tumor-suppressive properties with the same rigor applied to their protumoral roles.

In in vitro models, MSC-derived secretomes have demonstrated antitumor effects by disrupting key metabolic and survival pathways in GBM cells. Several studies have reported mechanisms involving apoptosis induction, reduced proliferation, and alterations in tumor bioenergetics. For example, Prateeksha et al. (2023) showed that dental pulp–derived MSCs decreased proliferation and metabolic activity of U87MG cells through increased ROS generation [161]. Similarly, Goodarzi et al. (2020) found that bone marrow–derived MSCs exerted direct cytotoxicity against C6 glioma cells by reducing proliferation and promoting apoptosis [122]. In murine orthotopic models, these MSCs decreased tumor size, cellular density, and invasiveness, resulting in significantly prolonged survival [122]. These findings suggest that specific MSC subpopulations can exert direct inhibitory effects on GBM progression.

Beyond direct cell–cell interactions, a substantial component of MSC antitumor activity has been attributed to paracrine mechanisms that interfere with essential survival programs of GBM cells. Notably, the secretome of adipose-derived MSCs has been shown to suppress late stages of tumor autophagy through activation of mTORC1 and reduction in TFEB nuclear translocation, ultimately decreasing tumor viability and enhancing apoptosis [162]. A similar effect was reported for conditioned medium from umbilical cord–derived MSCs, which increased proapoptotic gene expression and inhibited survival pathways in U87MG cells [163]. Additionally, bone marrow–derived MSCs have been shown to secrete proteins with antiproliferative and anti-invasive activity against GBM cells [145].

Collectively, these studies highlight that the functional state and tissue origin of MSCs critically shape the composition of their secretome and its impact on tumor cells. Importantly, the antitumor paracrine effects are not universal and coexist with substantial evidence of protumoral MSC activities discussed earlier. This duality underscores the need for a nuanced, context-specific interpretation of MSC behavior within the GBM microenvironment.

## 5. Cell-Based Therapeutic Strategies

### 5.1. Unmodified Cell Therapies: Rationale and Preclinical Evidence

#### 5.1.1. Unmodified Cell Therapies with TAMs

The concept of using “unmodified” macrophages as a cell therapy in cancer usually refers to autologous or allogeneic monocytes/macrophages that are not genetically engineered but may be differentiated or functionally polarized ex vivo before infusion. This strategy aims to exploit intrinsic macrophage properties, tumor infiltration, phagocytosis, antigen presentation, and functional plasticity, without the added complexity of viral vectors or gene-editing platforms. In principle, tumor-homing macrophages could be adoptively transferred as living drugs to exert direct cytotoxicity, remodel the TME, and orchestrate downstream T-cell responses. Historically, this idea has been explored in a small number of early-phase clinical trials in more common malignancies, where autologous monocytes were isolated, differentiated into macrophages, and activated ex vivo (typically with IFN-γ) before reinfusion into patients. These studies demonstrated that adoptive transfer of ex vivo–generated cytotoxic macrophages is technically feasible and generally safe, with occasional evidence of disease stabilization or minor tumor regression, but without consistent, durable objective responses [164]. Thus, while they provided proof-of-principle that macrophages can be used as effector cells in adoptive immunotherapy, they also highlighted the challenges of achieving sufficient in vivo persistence, maintaining a proinflammatory phenotype within an immunosuppressive TME, and scaling up manufacturing to clinically meaningful doses.

In GBM, adoptive macrophage-based therapies remain at a preclinical stage. Recent murine studies have shown that ex vivo–activated macrophages, when adoptively transferred, can infiltrate orthotopic GBM, suppress tumor growth, and reconfigure the TME toward a more inflamed state with increased CD8^+^ T-cell infiltration [165]. However, available data is scarce and indicates that truly unmodified macrophages (i.e., without ex vivo activation, drug loading, or genetic engineering) have been used only sparsely and with limited antitumor impact in human cancers, and have not been systematically evaluated in GBM [166]. Nevertheless, the underlying biological rationale remains compelling: macrophages are among the most abundant immune cells in GBM, they readily infiltrate hypoxic and perivascular niches that are poorly accessible to T cells, and they possess inherent phagocytic and antigen-presenting functions that could, in theory, be harnessed for therapy. The main obstacles are their pronounced plasticity and susceptibility to re-education by the GBM microenvironment, which can rapidly skew them back toward an immunosuppressive, tumor-promoting phenotype, as well as practical constraints related to cell sourcing, standardization, and large-scale GMP production. For these reasons, current development in the field is moving toward more controlled strategies, such as ex vivo–reprogrammed or engineered macrophages, while unmodified macrophage transfer is better viewed as an important conceptual and historical foundation rather than a mature therapeutic option at this stage.

#### 5.1.2. Unmodified Cell Therapies with MSCs

The early rationale for using unmodified MSCs in GBM therapy centered on their inherent tropism toward tumor regions and immunoregulatory properties. As discussed previously, MSC migration has been demonstrated both in vitro and in vivo and is partly mediated by TGF-β acting through the CD105 receptor, inducing lamellipodia formation and facilitating cellular locomotion [167]. In addition to canonical chemotactic cues such as SDF-1 and TGF-β [104], CCL5 has been identified as an alternative chemoattractant acting through CCR5, with MSC migration enhanced under conditions of intratumoral hypoxia [168]. MSCs also offer a theoretical advantage for allogeneic therapies due to their immune-privileged phenotype, characterized by low or absent expression of MHC class II molecules, enabling evasion of immune recognition and potentially circumventing the need for immunosuppression in clinical applications [169].

However, despite these favorable properties, accumulated preclinical evidence reveals substantial controversy and translational obstacles. The dual biological role of MSCs in GBM, where they can either attenuate tumor proliferation and invasion or enhance tumor growth, ECM remodeling, hybrid cell formation, and immunosuppressive circuits, results in inconsistent outcomes across studies. This variability reflects dependence on tissue origin, nature of interaction (paracrine vs. direct contact), experimental model, and degree of tumor education. Collectively, these findings indicate that unmodified MSCs possess an intrinsically unstable therapeutic profile and remain difficult to standardize for clinical implementation.

These limitations have driven the development of alternative strategies, including genetically modified MSCs, use of MSC-derived secretomes, cellular vectors for targeted drug delivery, and combinatorial approaches designed to impose stable antitumor functions and overcome the intrinsic contextual sensitivity of MSC biology.

### 5.2. Genetically Engineered Cell Therapies

Genetic engineering of immune and stromal cells has emerged as one of the most dynamic frontiers in cancer immunotherapy. In GBM, the unique capacity of macrophages and MSCs to home to tumor sites, integrate into the TME, and engage in sustained crosstalk with neoplastic and immune cells makes them particularly attractive candidates for “living drugs.” Instead of acting solely as passive components of the TME, these cells can be reprogrammed to deliver therapeutic payloads, reshape local immunity, and disrupt tumor-supportive circuits. Current engineering strategies broadly fall into three categories: (1) receptor-level rewiring, such as the introduction of chimeric antigen receptors (CARs) to confer tumor antigen specificity; (2) payload delivery, including enforced expression of cytokines, chemokines, or costimulatory molecules to boost antitumor immunity; and (3) enzyme–prodrug or suicide gene systems, in which engineered cells locally convert a systemically administered prodrug into its active cytotoxic form or undergo controlled cell death to limit toxicity. Although most of these approaches remain at the preclinical stage in GBM, accumulating evidence indicates that genetically engineered macrophages and MSCs can overcome some of the limitations of their unmodified counterparts and may provide a versatile platform for combinatorial therapies with radiotherapy, chemotherapy, or immune checkpoint blockade [170].

#### 5.2.1. Genetically Engineered Macrophages

Macrophages are particularly compelling targets for genetic engineering in gliomas because of their robust tumor tropism, capacity for phagocytosis and antigen presentation, and central role in shaping the immunosuppressive TME. In their unmodified state, TAMs frequently adopt protumoral phenotypes; however, when reprogrammed or armed with synthetic receptors and payloads, they can be converted into potent effectors of antitumor immunity [56].

One of the most advanced macrophage-based strategies in GBM involves CAR-modified macrophages (CAR-M). In a landmark preclinical study, Chen et al. engineered intracavitary macrophages to express a CD133-specific CAR directed against GSCs using NP-mediated gene transfer in a postoperative GBM model. These CD133-CAR macrophages exhibited enhanced, antigen-specific phagocytosis of GSCs, promoted local T cell priming, and improved survival, indicating that CAR-M can simultaneously mediate direct tumor clearance and recondition the TME toward an immunostimulatory state [171]. Recent reviews on next-generation brain cancer cell therapies further highlight CAR-M for their high tumor infiltration, low systemic toxicity, and capacity to reverse local immunosuppression, positioning them as a promising complement or alternative to CAR-T approaches in GBM [172].

A remarkable recent advance is presented in a 2025 study in which researchers used enucleated MSCs as vehicles for in situ delivery of CAR-encoding plasmids, thereby converting GA-TAMs into CAR-M directly within the tumor. This approach bypasses the need for ex vivo cell manufacturing and produced sufficient numbers of CAR-M in gliomas in vivo. Following treatment, especially when combined with blockade of the “don’t eat me” signal CD47, mice bearing orthotopic GBM experienced almost complete tumor suppression and markedly prolonged survival [173].

Beyond CARs, macrophages and myeloid cells have been engineered to deliver cytokines in situ. Canella et al. generated bone marrow–derived myeloid cells engineered to express IL-2 and showed that their intratumoral or systemic administration in glioma-bearing mice reprogrammed the TME, increasing cytotoxic T-cell infiltration, reducing immunosuppressive populations, and prolonging survival [174]. This work supports the concept that engineered myeloid cells can act as localized cytokine factories, overcoming the pharmacokinetic and toxicity limitations of systemic cytokine therapy while exploiting the natural myeloid tropism for gliomas.

Foundational platforms have also demonstrated that lentivirally engineered macrophages can persist in solid tumor models and constitutively express therapeutic proteins, including inflammatory cytokines such as Il-12 or costimulatory ligands, with sustained antitumor activity and evidence of TME remodeling [175]. Although most studies to date have focused on peripheral solid tumors, these technologies are readily adaptable to GBM, and several groups now explicitly propose their application to brain tumors, particularly in combination with radiotherapy or checkpoint blockade [172]. A key example is described in the study presented by Gardell J et al. (2020), in which human monocyte-derived macrophages were genetically modified (GEMs) to secrete a bispecific T-cell engager (BiTE) targeting the GBM-specific antigen EGFRvIII [176]. These BiTE-secreting macrophages induced robust activation, proliferation, degranulation and cytotoxic responses in T cells, leading to antigen-dependent killing of glioma cells in vitro and reducing early tumor burden in both subcutaneous and intracranial GBM xenograft models. The antitumor effect was further enhanced when these macrophages were dual-engineered to also secrete IL-12, underscoring the potential of GEMs as localized “T-cell engager factories” that can overcome limitations of systemic BiTE delivery, such as poor brain penetration or systemic toxicity.

Engineered macrophage platforms have begun to explore enzyme–prodrug systems, constitutive cytokine expression, and sustained secretion of immunomodulatory molecules as means to concentrate therapy within the TME while minimizing systemic toxicity. A notable example is the use of photochemical internalization (PCI) to deliver a suicide-gene, the cytosine deaminase (CD) gene, into macrophages. In the study by Romena et al., PCI-transfected macrophages were able to convert the non-toxic prodrug 5-fluorocytosine (5-FC) into the cytotoxic 5-fluorouracil (5-FU), which exerted a strong bystander killing effect on neighboring glioma cells in co-culture [177].

Collectively, these developments illustrate how GEMs can be transformed from passive accomplices of GBM into active therapeutic agents. By combining tumor-specific recognition (via CARs), localized cytokine or BiTE delivery, and potentially enzyme–prodrug systems, engineered macrophages offer a multifaceted platform to attack gliomas, remodel the TME, and synergize with existing therapies. That said, key challenges remain before these approaches can be translated into clinical practice such as efficient and reproducible manufacturing, stable expression and functionality of the engineered payload, control of off-target effects or toxicity, and demonstration of safety and persistence in the human brain.

#### 5.2.2. Genetically Engineered MSCs

In parallel, extensive work has focused on engineering MSCs to deliver pro-apoptotic agents, immunomodulatory cytokines, or therapeutic microRNAs into GBM. MSCs are particularly attractive because of their robust tumor tropism, immune evasiveness, and capacity to serve as cellular factories for sustained release of engineered payloads.

Multiple studies have used adenoviral, lentiviral, plasmid-based, and non-viral delivery systems to program MSCs for tumor suppression [178,179,180]. Non-viral strategies have gained momentum due to their scalability, reversibility, and improved safety profiles.

NP-mediated systems represent a particularly promising direction. In one example, NPs were used to deliver pro-apoptotic TRAIL genes to MSCs, enabling tumor-localized apoptosis without impairing MSC viability [181]. In another, iron oxide NPs and plasmids encoding HSV-TK were co-delivered to MSCs to induce CX43 overexpression and suicide gene therapy, exploiting gap-junction transfer to kill neighboring tumor cells [182]. These approaches convert inherent tumor-supportive traits into therapeutically exploitable mechanisms.

Other platforms target the immunosuppressive niche. Anucleated MSCs carrying CAR-macrophage plasmids can undergo apoptosis within tumors and transfer CAR constructs to local macrophages, enabling in situ generation of CAR-M and enhancing M1 polarization and T-cell infiltration [173]. Additional models incorporate cytokines such as CXCL10, IL-12, nCD47-SLAMF7, PD-1, or IL-18-Fc to promote recruitment of cytotoxic T cells, repolarization of macrophages, or amplification of antitumor inflammatory responses [179,183,184,185].

Finally, adenoviral vectors have been used to deliver tumor-suppressive microRNAs such as miR-124 and miR-4731-5p into MSCs, resulting in reduced proliferation, increased apoptosis, and cell-cycle arrest in GBM models [178,180].

In combination, engineered macrophages and MSCs represent complementary, modular toolkits capable of reshaping the GBM microenvironment at multiple levels: tumor killing, immune activation, stromal remodeling, and metabolic disruption. Their integration with synthetic biology, non-viral engineering, and controlled delivery technologies positions them as leading platforms in the emerging field of programmable cell-based therapies for malignant gliomas.

### 5.3. Cellular Carriers for Oncolytic Viruses and Biotherapeutics

**Figure 3:**
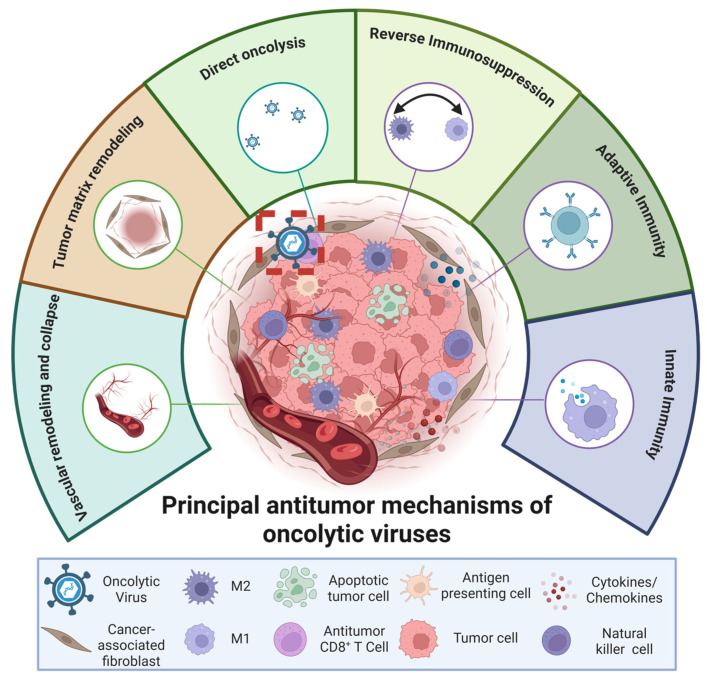
Core antitumor mechanisms of oncolytic viruses (OVs). Oncolytic viruses suppress tumor growth through multiple, complementary mechanisms, including the selective infection and lysis of malignant cells, activation of innate and adaptive immune responses, reprogramming of immunosuppressive cell populations and ECM components, and modulation of tumor angiogenesis within the TME. Created in BioRender. Ibarra, L. (2026) https://BioRender.com/tetlisw (accessed on 12 December 2025).

The use of living cells as carriers for oncolytic viruses (OVs) and other biotherapeutic agents has gained considerable attention as a strategy to overcome major barriers in virotherapy, including viral neutralization in circulation, limited intratumoral delivery, and rapid clearance by the immune system (Figure 3). Macrophages, in particular, have emerged as promising cellular vehicles because of their intrinsic tumor-homing capacity, phagocytic competence, and natural ability to infiltrate hypoxic and therapy-resistant tumor regions. In breast cancer models, macrophages loaded ex vivo with the oncolytic herpesvirus HSV1716 (M-HSV1716) displayed enhanced therapeutic efficacy following systemic administration, achieving significant tumor regression and survival benefit with a 100-fold lower viral dose compared with free virus delivery. These findings indicate that macrophage-mediated transport can improve OV biodistribution while reducing systemic toxicity [186]. A recent review further emphasized that macrophages may function effectively as OV “cell carriers” due to their tumor tropism, although their potential to inactivate the virus or repolarize toward pro-tumoral (M2-like) states remains a critical limitation for successful virotherapy [187].

In GBM, however, direct use of macrophages as OV carriers remains largely unexplored. Most published studies instead highlight the role of macrophages and microglia as modulators of OV activity within the TME, rather than as active delivery vehicles. Importantly, virotherapy itself can remodel non-neoplastic stromal populations. Given that GBM lacks canonical CAFs, pericytes co-expressing FAP and PDGFRβ have been identified as the principal CAF-like stromal cells in this tumor type [188]. A modified oncolytic adenovirus (ICOVIR15; ∆24-E1A + RGD-fiber) was shown to infect and selectively deplete these FAP^+^/PDGFRβ^+^ stromal cells in addition to GBM cells, thereby disrupting key supportive compartments of the TME [188]. This work underscores that the efficacy of virotherapy depends not only on tumor-cell lysis but also on its capacity to modulate pro-invasive and immunosuppressive stromal niches.

Furthermore, recent preclinical and clinical observations demonstrate that OV administration can reprogram the GBM immune milieu. Oncolytic viruses consistently promote influx and/or repolarization of macrophages toward proinflammatory M1-like states, stimulate effector T-cell recruitment, and enhance local immunostimulatory signaling, contributing to improved antitumor responses [189]. Despite these insights, no published studies to date have demonstrated successful therapeutic delivery of OVs using macrophages as carriers in orthotopic GBM models, underscoring a critical knowledge gap. Advancing this approach will require optimization of viral loading, verification of macrophage trafficking into the brain, efficient intratumoral viral release, and robust testing in clinically relevant GBM systems.

In this context, it is important to recognize that not all viruses interacting with the TME elicit immunostimulatory effects. Emerging experimental evidence indicates that human cytomegalovirus (HCMV) may actively contribute to GBM progression through mechanisms of oncomodulation and, potentially, oncogenesis. Notably, Guyon et al. (2024), through the analysis of human tumor specimens and complementary experimental models, demonstrated that HCMV presence is associated with the activation of pro-tumorigenic signaling pathways, extensive remodeling of the TME, and the establishment of a profoundly immunosuppressive milieu in GBM [190].

Consistently, El Baba et al. (2023) provided mechanistic evidence showing that HCMV can directly infect macrophages and reprogram them from a pro-inflammatory (M1) phenotype toward an M2/TAM-like state characterized by immunosuppressive and tumor-supportive functions, both in vitro and in vivo [191].

Collectively, these findings introduce a critical consideration for virus-based therapeutic strategies in GBM. While oncolytic virotherapy has the potential to promote macrophage repolarization toward antitumoral phenotypes, certain persistent viruses, such as HCMV, may exert the opposite effect by reinforcing immunosuppression within the TME. Consequently, virus–macrophage interactions emerge as a key determinant of therapeutic efficacy and should be carefully evaluated when designing therapeutic approaches that integrate viral platforms and myeloid cells for GBM treatment.

MSCs have also been widely explored as carriers for oncolytic adenoviruses in GBM therapy. Several studies have shown that MSC-mediated OV delivery can enhance viral dispersion, tumor penetration, and immunomodulation. Mahasa et al. used an integrated mathematical–experimental framework to demonstrate that MSCs loaded with an oncolytic adenovirus accelerated tumor lysis and improved tumor control compared with free virus administration [192]. Zhang et al. engineered umbilical cord-derived MSCs to carry a doxycycline-inducible adenovirus encoding IL-24 and endostatin, enabling controlled viral replication within MSCs. In glioma-bearing mice, these MSC carriers released the virus in a regulated manner, inducing apoptosis and suppressing angiogenesis [193].

More advanced systems have been designed to restrict viral release to the tumor site. For example, MSCs engineered to express GRP78 together with an inducible E1B55K adenoviral system exhibited controlled OV production upon tumor infiltration, ensuring spatially confined viral amplification [194]. Another recent study employed MSCs loaded with Ad5-Ki67/IL-15, achieving potent oncolytic activity at low viral doses and increased macrophage infiltration within GBM tumors [195]. In a clinically relevant approach, Shimizu et al. isolated bone marrow-derived MSCs from patients with recurrent GBM and demonstrated that these autologous MSCs efficiently homed to GBM xenografts and delivered the Delta-24-RGD adenovirus, validating the feasibility of patient-derived MSC carriers [196].

Collectively, these findings highlight the emerging potential of macrophages and MSCs as cellular vehicles for oncolytic virotherapy and biotherapeutic agents. While MSC-based strategies are substantially more advanced in GBM, macrophage carriers remain conceptually appealing yet technically underdeveloped. Bridging this gap will require rigorous optimization of loading strategies, carrier stability, trafficking behavior in the brain, and therapeutic release mechanisms. Nevertheless, the convergence of cellular delivery systems with next-generation OVs offers a promising frontier for enhancing GBM treatment efficacy through targeted, localized, and multimodal antitumor activity.

### 5.4. Combined Approaches (Cell Therapy Plus Radiotherapy, Chemotherapy, Immunotherapy)

#### 5.4.1. Macrophage-Based Combinatorial Strategies

Combining macrophage-based cellular therapies with radiotherapy (RT), chemotherapy, photodynamic therapy (PDT) or immune checkpoint inhibition has gained interest as a rational means to overcome limitations inherent to each modality, which are well established for brain tumors. GBM is characterized by profound immunosuppression, antigenic heterogeneity, and restricted intratumoral distribution of therapeutics, factors that blunt the efficacy of both adoptive cell therapy and conventional treatments when administered independently. Preclinical evidence increasingly supports the hypothesis that macrophage-directed therapies may synergize with RT or cytotoxic drugs: radiation and chemotherapy induce immunogenic stress, increase neoantigen exposure, and promote the release of DAMPs, thereby enhancing the proinflammatory activation of reprogrammed macrophages and facilitating antigen presentation and recruitment of CD8^+^ T cells. Extending this rationale, macrophage-based strategies have also been explored in combination with PDT, a modality that generates localized ROS upon light activation but is limited by poor photosensitizer delivery and restricted light penetration in brain tumors [197].

In this regard, macrophages have been investigated as cellular vectors for PDT, functioning both as carriers of photosensitizers and as active modulators of the TME. Importantly, the strategies described in the literature principally employ circulating monocytes or monocyte-derived macrophages that are isolated or generated ex vivo, loaded with photosensitizing agents, and adoptively reintroduced to exploit their intrinsic recruitment to chemokine-rich, hypoxic, and necrotic regions of GBM [197]. Resident brain macrophages (microglia) are generally not used for ex vivo loading approaches, although they may take up photosensitizers indirectly in situ and contribute to local effects. The tumor-homing behavior invoked here therefore refers specifically to the migratory capacity of these adoptively transferred, photosensitizer-loaded cells rather than to all macrophage populations indiscriminately.

Phenotype and tropism are related but distinct properties: TAM-like (M2-skewed) macrophages often display greater retention and survival within the GBM niche, whereas M1-polarized cells can robustly home to inflammatory niches but may differ in persistence and functional fate after arrival. Mechanistic studies show that light activation of macrophage-delivered photosensitizers can produce localized cytotoxicity and concurrently trigger proinflammatory reprogramming of the TME, thereby enhancing antigen availability and immune signaling—effects consistent with an in situ vaccination paradigm [197].

Alternative, entirely in vivo “vaccination” concepts, where naïve circulating monocytes are programmed systemically by administrating nanoparticles, photosensitizers, or immunomodulators that are preferentially taken up in circulation, are promising but remain experimental. Compared with ex vivo loading, in vivo monocyte programming could simplify logistics but currently offers less control over payload dose, cell phenotype, and biodistribution, and poses additional challenges for specificity and safety in the GBM context. Taken together, macrophage-mediated PDT is a conceptually attractive approach that requires continued optimization of cell source, loading method, polarization control, and delivery strategy to maximize tumor selectivity and durable antitumor immunity.

In orthotopic GBM models, combinatorial interventions incorporating therapeutic macrophages have yielded more potent antitumor effects than monotherapies. Fan et al. (2025) reported that adoptive transfer of reprogrammed macrophages, when combined with low-dose irradiation and PD-1 blockade, produced superior tumor control and prolonged survival in orthotopic GBM [165]. This regimen induced a pronounced immunologic reconfiguration of the TME, characterized by increased CD8^+^ infiltration and a shift toward a proinflammatory cytokine milieu, illustrating how RT or checkpoint inhibition can provide critical sustaining signals for the antitumor phenotype of adoptively transferred macrophages. Similarly, Sun et al. (2025) demonstrated that macrophage–drug conjugates used as delivery vehicles showed improved biodistribution and enhanced local efficacy when combined with RT or chemotherapy, indicating that combinatorial regimens may be both synergistic, yielding stronger tumoricidal effects and permissive, allowing lower systemic dosing of cytotoxic agents [198].

Translation of macrophage-centered combinations to the clinic, however, remains at an early stage. The first CAR-macrophage product to enter human testing (CT-0508, anti-HER2) has demonstrated manufacturability and acceptable safety in patients with HER2^+^ solid tumors and is currently being evaluated in combination with pembrolizumab (NCT04660929). Yet, this trial does not include GBM, and no published clinical studies to date have investigated adoptive macrophage transfer or CAR-M therapy combined with RT, chemotherapy, or immunotherapy in GBM patients. Instead, early-phase trials focusing on checkpoint inhibitors such as relatlimab (anti–LAG-3) alone or combined with nivolumab (NCT02658981; NCT03493932) underscore the broader effort to enhance immunotherapy efficacy in GBM, though these approaches do not specifically integrate macrophage-based therapeutics.

In light of these findings, several conceptual and practical challenges must be recognized when advancing macrophage-based combinatorial approaches in GBM. TAM plasticity can lead to undesired repolarization toward tumor-supportive phenotypes; delivery barriers imposed by the BBB may limit cell homing; and combined immunostimulatory strategies risk neuroinflammation in a highly sensitive organ. Furthermore, GBM lacks reliable biomarkers to guide patient selection or optimize treatment sequence and dosing. As emphasized in recent translational recommendations [199], future development should prioritize predictive orthotopic models, detailed immune correlatives in resected tissue, and inducible or safety-switch-equipped cellular platforms to enable controlled activation in clinical combination trials.

#### 5.4.2. MSC-Based Combinatorial Strategies

Genetically engineered MSCs offer complementary opportunities for combination therapy, functioning not only as delivery vectors but also as amplifiers of cytotoxic, apoptotic, or immunomodulatory interventions. Because unmodified MSCs display highly variable behavior in GBM, ranging from antitumor effects to the promotion of invasion, ECM remodeling, or immunosuppression, engineering and combinatorial regimens aim to override this instability and redirect MSCs toward consistent therapeutic outputs.

One major avenue involves enhancing apoptosis through combined MSC-derived TRAIL expression and chemotherapeutic sensitization. In Coccè et al. (2020), paclitaxel-preconditioned TRAIL-MSCs exhibited increased TRAIL secretion and elevated release of antitumor mediators, representing a scalable means to boost efficacy without increasing genetic complexity [200]. However, the study remained limited to in vitro settings and did not examine whether paclitaxel alters MSC migratory capacity, an essential consideration for in vivo applications [200]. Han et al. (2019) further demonstrated synergistic apoptosis via co-application of TRAIL-secreting MSCs and AMPK inhibition (Compound C) [201]. Because AMPK activation can confer metabolic resilience to GBM cells, its inhibition enhanced TRAIL/DR5-mediated apoptosis, though potential activation of compensatory survival pathways and the absence of in vivo validation highlight remaining uncertainties [201].

Other studies have explored natural compounds as adjuvants to MSC therapy. Nascimento et al. (2023) showed that coadministration of MSCs and agathiflavone increased GBM apoptosis by modulating STAT3 signaling within MSCs, reprogramming them into a more antitumor phenotype [202]. Despite its conceptual simplicity and low cost, the mechanism of synergy, whether secretome-mediated, contact-dependent, or purely additive, remains incompletely defined, and the lack of in vivo confirmation limits translational interpretation.

Combinations of microRNAs and chemotherapy represent another promising direction. Sharif et al. (2018) demonstrated that miR-124–loaded MSCs decreased GBM proliferation and enhanced TMZ sensitivity by regulating STAT3 and cell-cycle pathways [178]. However, microenvironmental effects of miR-124 were not evaluated. Complementing this approach, Chang et al. (2020) employed MSCs expressing CD to convert 5-fluorocytosine into 5-fluorouracil, producing a robust cytotoxic effect that synergized with TMZ both in vitro and in vivo [203]. This study underscores the utility of MSC-based enzyme–prodrug therapies as combinatorial sensitizers capable of reducing tumor proliferation more effectively than monotherapies.

MSC-based strategies also intersect increasingly with immunotherapy. As described in Section 5.2.2., Zhou et al. (2025) developed an anucleated MSC platform that delivers CAR-M plasmids to the tumor niche, allowing resident macrophages to phagocytose MSC fragments and generate CAR-M in situ [173]. When combined with CD47 blockade, this approach achieved complete tumor suppression and extended survival by 83%, illustrating the potential of MSC–macrophage synergies to amplify immune-mediated tumor clearance. Similarly, Mao et al. (2023) showed that MSCs engineered to overexpress CXCL10 and Nrf2 enhanced immune infiltration and cytotoxic activity within GBM, while also increasing responsiveness to immune checkpoint blockade [179]. Although promising, potential long-term survival advantages conferred by Nrf2 signaling in GBM must be considered.

Collectively, these findings illustrate that MSC-based therapies can function as delivery systems, sensitizers, and immune modulators within integrated therapy regimens. However, similar to macrophage-based combinations, problems persist, including inconsistent MSC behavior among GBM models, insufficient in vivo validation for numerous techniques, the possibility of immunosuppressive feedback, and the necessity for meticulous regulation of therapeutic payload release. Future research should incorporate orthotopic GBM models, real-time lineage tracing, and inducible genetic circuits to guarantee safe and sustained synergy with standard-of-care and novel therapies.

### 5.5. Cell-Derived Delivery Platforms

#### 5.5.1. EVs from Macrophages and MSCs: Classification and Cargo

EVs are natural carriers of molecular information that mediate both short- and long-range intercellular communication. In GBM, EVs derived from tumor-infiltrating macrophages and MSCs have emerged as key regulators of the TME, influencing tumor proliferation, invasion, angiogenesis, and immune modulation. EVs are broadly classified into small EVs—including exosomes—and large EVs, such as microvesicles and apoptotic bodies, according to their biogenesis, size, and molecular composition [204]. Among these populations, exosomes are the most extensively characterized and are defined as lipid bilayer–enclosed vesicles of approximately 50–200 nm in diameter, displaying a characteristic cup-shaped morphology under electron microscopy. They typically express markers such as CD63, TSG101, and HSP70, while lacking endoplasmic reticulum proteins such as calnexin, which is commonly used as a negative control [205]. Their cargo comprises proteins, nucleic acids (mRNAs and microRNAs), and lipids that collectively support intercellular signaling and functional reprogramming [206].

**Figure 4:**
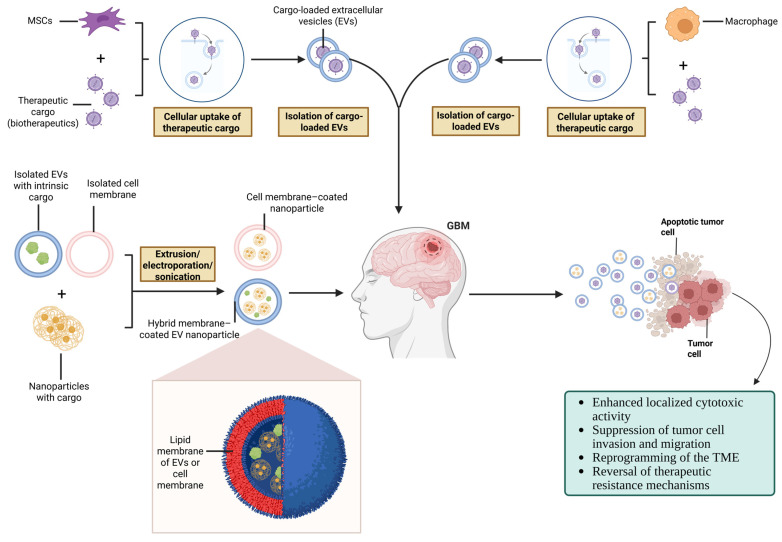
Engineering strategies for extracellular vesicle (EV)-based and cell-based therapeutic delivery in GBM. Schematic overview of cell-mediated and cell-free approaches to generate cargo-loaded EVs and cell membrane-based therapeutics for GBM therapy. Therapeutic payloads can be internalized by donor cells (e.g., MSCs or macrophages), followed by isolation of EVs naturally loaded with bioactive cargo. Alternatively, isolated EVs can be post-loaded using physical methods such as extrusion, electroporation, or sonication, or combined with cell membranes to generate hybrid-coated EV–nanoparticle systems. Following administration, engineered EVs accumulate within the GBM microenvironment and deliver their cargo to tumor and stromal cells, resulting in enhanced localized cytotoxicity, inhibition of invasion and migration, reprogramming of the TME, and reversal of therapeutic resistance. Created in BioRender. Ibarra, L. (2026) https://BioRender.com/xe0veot (accessed on 12 December 2025).

In the context of GBM, macrophage-derived EVs display functionally distinct profiles depending on the polarization state of the donor cell. EVs released by M1-polarized macrophages are enriched in antitumor microRNAs, such as miR-150, which can be transferred to glioma cells and suppress proliferation and invasion by targeting matrix-remodeling genes including *MMP16* [207]. In contrast, EVs derived from M2-like macrophages tend to carry immunosuppressive and tumor-promoting factors, thereby reinforcing GBM progression and immune evasion. This functional dichotomy highlights the importance of macrophage activation state in shaping EV cargo and biological impact, and it provides a rationale for selectively exploiting or engineering M1-derived EVs as antitumor nanocarriers (Figure 4).

MSCs also communicate extensively through EV secretion, and MSC-derived EVs exhibit marked heterogeneity in size, molecular composition, and biological function. Proteomic analyses have revealed enrichment of proteins involved in ATP and NAD binding, wound healing, lipid metabolism, and PI3K–AKT and VEGF signaling, as well as regulators of T-cell activation, Rap1/Ras signaling, and NF-κB pathways. Among the most abundant proteins identified are SYK, BTK, EGFR, and ICAM-2 [205]. At the nucleic acid level, MSC-derived EVs carry a broad repertoire of microRNAs that target oncogenes, tumor suppressors, and signaling pathways associated with proliferation, migration, and therapy resistance, thereby exerting context-dependent protumoral or antitumoral effects [184].

From a therapeutic perspective, MSC-derived EVs have attracted substantial interest as acellular delivery platforms. Compared with direct MSC administration, EV-based approaches offer several advantages, including enhanced biosafety, improved stability, lack of aneuploidy, and reduced immunogenicity, while preserving the ability to transport bioactive cargo across biological barriers such as the BBB [205]. In GBM models, MSC-derived EVs have been successfully loaded with microRNAs, suicide gene systems, cytokines, and immunomodulatory molecules, resulting in reduced tumor proliferation, increased apoptosis, remodeling of the TME, and prolonged survival in vivo.

The breadth and consistency of these findings are summarized in Table 3 and Table 4, which compiles representative studies employing MSCs or MSC-derived EVs and TAMS and TAMs-derived EVs as delivery platforms for therapeutic payloads in GBM models. Across diverse experimental settings, including in vitro systems and orthotopic in vivo models, EV-mediated delivery of microRNAs (e.g., miR-124, miR-133b, miR-142-3p), cytokines (IL-12, IL-18-Fc), immune checkpoint modulators (PD-1), and enzyme–prodrug systems (CD-UPRT) consistently resulted in suppression of tumor growth, induction of apoptosis or cell-cycle arrest, immune reprogramming of the TME, and improved survival, without evidence of MSC-associated tumorigenicity (Table 3).

Consequently, EVs originating from macrophages and MSCs represent a highly adaptable, cell-based delivery system in GBM. Their classification, molecular composition, and functional impacts are closely associated with the phenotype and origin of the donor cell (M1 versus M2 macrophages; naïve versus GA-MSCs), which subsequently dictates whether they promote tumor growth or facilitate anticancer responses. Leveraging this axis via enhanced donor-cell conditioning or genetic modification of EV-producing cells could facilitate the development of advanced EV-based therapeutics that can traverse the BBB, reshape the GBM microenvironment, and deliver targeted molecular payloads with diminished systemic toxicity.

#### 5.5.2. Engineering EVs for Targeted Payload Delivery

As previously mentioned, EVs have become particularly appealing vehicles for targeted therapeutic administration in GBM, due to their inherent biocompatibility, stability in circulation, and capacity to traverse complex biological barriers, including the BBB [216]. EVs—comprising exosomes, microvesicles, and apoptotic bodies—facilitate the normal transit of RNA species, proteins, lipids, and metabolites among cells. Their molecular payload can be systematically designed to convey mRNA, siRNA/miRNA, therapeutic proteins, or small molecules to specific target areas. Engineering methodologies often adhere to two complimentary strategies: (i) genetically altering donor cells to enable the endogenous packaging of therapeutic payloads into secreted EVs, or (ii) exogenously loading separated EVs using chemical, physical, or electroporation techniques. Both technologies facilitate the creation of vesicles with improved tumor targeting (e.g., by modified surface ligands) and refined intracellular trafficking for therapeutic efficacy. Nonetheless, the domain acknowledges that electric vehicle engineering necessitates stringent methodological criteria. The MISEV guidelines underscore the necessity for thorough characterization of EV preparations, encompassing size and concentration via NP tracking analysis or high-resolution flow cytometry; validation of canonical markers such as CD9, CD63, and CD81; and the elimination of soluble contaminants or protein aggregates [217]. Moreover, any engineering step, whether donor-cell transfection, expression of membrane-anchored targeting peptides, or RNA electroporation, must be accompanied by direct evidence that the therapeutic cargo is truly incorporated into EVs, remains structurally intact, and retains biological activity after purification. These requirements underscore a central principle: EV-based therapies are only as reliable as the stringency of their production and characterization pipelines.

**Figure 5:**
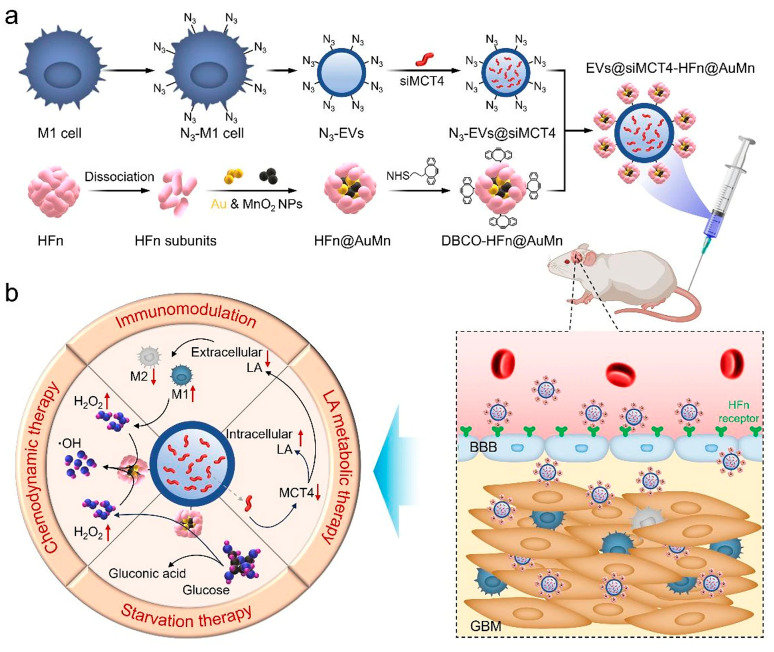
Engineering and therapeutic mechanism of macrophage-derived EVs for multimodal GBM treatment. (**a**) Schematic illustration of the fabrication process of M1 macrophage-derived EVs loaded with siMCT4 and functionalized with heavy-chain ferritin (HFn)-based Au/MnO_2_ nanostructures via bioorthogonal click chemistry, generating EVs@siMCT4-HFn@AuMn. (**b**) Proposed mechanism of action following systemic administration, highlighting efficient blood–brain barrier (BBB) traversal mediated by HFn receptor interactions, intratumoral accumulation, and combined therapeutic effects. Directional arrows denote molecular transport, catalytic reactions, and intracellular trafficking pathways. Red upward arrows (↑) indicate increased levels or activation (e.g., intracellular lactate accumulation, reactive oxygen species (ROS) generation, H_2_O_2_ production, and M1 macrophage polarization), whereas red downward arrows (↓) indicate decreased levels or inhibition (e.g., extracellular lactate export, MCT4 expression, and M2 macrophage polarization). Circular arrows represent metabolic conversion processes, including glucose oxidation and gluconic acid formation, leading to metabolic starvation. Together, this platform integrates metabolic interference, redox-based cytotoxicity, and immune reprogramming to suppress GBM growth. Reproduced with permission from [218], Journal of Nanobiotechnology (2025), open-access article distributed under the Creative Commons Attribution License (CC BY 4.0).

Recent advances in EV engineering illustrate the translational potential of macrophage- and MSC-derived vesicles for GBM therapy. In a notable example, Lu et al. (2025) developed M1-macrophage-derived EVs equipped with ferritin nanostructures and Au/MnO_2_ NPs to facilitate BBB penetration and deliver siMCT4 while generating reactive oxygen species within the tumor niche (Figure 5) [218]. This combinatorial strategy significantly reduced glioma growth in xenograft models, demonstrating that engineered EVs can co-deliver genetic and oxidative stress-inducing cues with high precision. Complementary work by Saito et al. (2021) showed that the molecular content of EVs mirrors the differentiation state of donor cells, with specific shifts in miRNA and proteomic profiles occurring during neural lineage commitment [219]. These findings highlight two fundamental insights for therapeutic design: donor cells can be programmed to selectively “package” desired nucleic acids or proteins into EVs, and molecular profiling of EV cargo can serve as a quality-control tool to predict functionality in neural or tumor tissues.

The importance of donor-cell identity is further reinforced by studies on GSC-derived EVs. Spinelli et al. (2018) demonstrated that proneural and mesenchymal GSC subtypes release vesicles with markedly distinct proteomes, membrane markers, and pro-angiogenic capacities [220]. Mesenchymal GSC-EVs, enriched in tetraspanins and endothelial-stimulatory proteins, drive aggressive vascular remodeling; proneural EVs display divergent uptake and signaling characteristics. This intrinsic heterogeneity parallels the practical challenges in EV engineering: the biological state of the donor cell shapes the vesicular landscape, influencing therapeutic efficacy, targeting specificity, and consistency across preparations. Therefore, EV design for GBM must consider not only the engineering modality but also the lineage, activation state, and transcriptional program of the donor cell.

MSC-derived EVs have gained particular attention as therapeutic vehicles due to their low immunogenicity, genomic stability, and favorable safety profile compared with MSCs themselves [205]. Proteomic analyses reveal that MSC exosomes contain proteins linked to ATP/NAD binding, wound repair, lipid metabolism, and PI3K–AKT and VEGF signaling, along with microRNAs involved in T-cell activation, Ras/Rap1 pathways, and NF-κB regulation. Because microRNAs can regulate oncogenes, tumor-suppressor pathways, and genes controlling GBM proliferation and migration [184], MSC-derived EVs naturally contain functionally potent regulatory molecules that can be redirected toward therapeutic benefit when engineered appropriately.

When MSCs are bioengineered to package defined therapeutics, whether microRNAs, suicide enzymes, immunomodulatory proteins, or gene-editing constructs, their EVs consistently exhibit antitumor activity across in vitro and in vivo GBM models. Reported effects include suppression of proliferation, reduction in migratory and invasive capacity, induction of apoptosis, enhanced chemosensitivity, decreased tumor burden, and improved survival. This engineered uniformity strategically eliminates the ambiguity associated with the dual native roles of MSCs and transforms them into predictable, programmable delivery vehicles whose therapeutic effects reflect engineered cargo rather than endogenous paracrine signaling. The use of purified EVs further minimizes risks of MSC-mediated immunosuppression or tumor-promoting interactions.

Despite this promise, several translational challenges remain such as standardizing EV yields and doses, establishing scalable clinical-grade purification protocols, ensuring reproducibility across donor batches, and verifying intracerebral biodistribution. Nonetheless, the collective evidence positions macrophage- and MSC-derived EVs, especially those rationally engineered with targeted nucleic acids, immune-modulating proteins, or proapoptotic factors, as next-generation delivery platforms with strong potential for GBM therapy.

### 5.6. Cell Membrane-Coated NPs

The concept of enveloping therapeutic platforms with natural cell membranes has swiftly emerged as a revolutionary method in nanomedicine, especially for central nervous system (CNS) diseases like GBM. Membrane-coated nanotechnologies utilize the intrinsic biocompatibility, immune-evasive characteristics, and tissue-targeting abilities of cellular membranes to address significant challenges that hinder traditional NP delivery to brain tumors, such as swift clearance by the reticuloendothelial system, inadequate intratumoral penetration, and limited transport across the BBB. Although several membrane sources have been explored, macrophage-derived membranes have gained special prominence for GBM and other CNS malignancies. Macrophages naturally home to inflammatory and hypoxic regions, can traverse the BBB under pathological conditions, and exhibit strong tropism toward glioma-associated chemokines—features that are retained, at least partially, in macrophage membrane coatings. These biological attributes endow macrophage-membrane-coated NP with superior capacity to navigate CNS barriers, evade immune recognition, and accumulate in tumor niches enriched in myeloid cells. While cell membrane-coated NPs (CM-NPs) constitute the most widely investigated application of this concept, membrane biomimicry has also been extended to hydrogels, hybrid vesicles, and implantable devices, all designed to enhance stability, immune evasion, and spatial targeting within the CNS [221,222,223]. A comprehensive body of work indicates that CM-NPs provide multiple advantages over traditional nanocarriers, including prolonged systemic circulation, reduced opsonization, improved biocompatibility, enhanced BBB permeability, and superior intratumoral accumulation [224]. For example, recent studies demonstrated that macrophage-derived membrane coatings markedly improve delivery efficiency of drug-loaded or RNA-loaded NPs into GBM tissue while minimizing off-target effects outside the brain [214,225]. Furthermore, these membranes can be engineered to incorporate adhesion ligands, chemokine receptors, or signaling proteins that enrich NP tropism for specific tumor niches or microenvironmental subdomains.

From a therapeutic perspective, macrophage membrane-coated NPs offer a compelling strategy for uniting the innate tumor-homing behavior of myeloid cells with controlled-release nanosystems. Their intrinsic ability to recognize inflammatory cues, infiltrate hypoxic tumor regions, and engage with stromal elements positions them as highly suitable vehicles for localized delivery of cytotoxic agents, immunomodulators, or therapeutic RNAs directly into the postsurgical tumor niches. This approach aligns with emerging concepts of “cell-inspired delivery,” where membrane biomimetics allow NPs to behave as functional surrogates of infiltrating immune cells.

Despite these promising attributes, important translational challenges remain. CM-NP manufacturing is constrained by high production costs, complex multistep preparation workflows (including membrane isolation, purification, and coating), and limited understanding of long-term biodistribution or immunological impact in humans. These factors currently hinder large-scale clinical deployment and underscore the need for standardized, GMP-compatible production pipelines.

In parallel, a rapidly expanding set of studies published between 2020 and 2025 demonstrate that MSC-derived membrane coatings confer major advantages to NP formulations, including improved pharmacokinetics, enhanced biocompatibility, immune evasion, and superior tumor targeting. MSC membrane-coated systems have been successfully applied in PDT [226], nuclear-targeted nanocarriers [227], CNS-targeting nanostructures [228], and biomimetic metal–organic frameworks [229]. Although macrophage- and dendritic cell-derived membranes have been used to coat NPs in GBM models, only one study has thus far employed MSC membrane-coated NP in GBM, despite their robust preclinical efficacy in other cancer and CNS-related scenarios. This study demonstrates the viability of utilizing MSC-derived membrane coatings in GBM treatment by creating a biomimetic nanoplatform that combines a genetically modified MSC membrane with a bioactive NP core for chemokine-directed radioimmunotherapy. The membrane component, originating from CCR2-overexpressing MSCs, operates as a directional “tactical tentacle” that utilizes radiation-induced CCL2 overexpression in gliomas for precise targeting. The NP core, consisting of diselenide-bridged mesoporous silica NPs (MSNs) coupled with anti-PD-L1 antibodies, facilitates X-ray–responsive drug release and improves radiosensitization. This hybrid method demonstrates the rational engineering of MSC membrane-coated NP to integrate tumor-targeting with immune checkpoint regulation in GBM [230]. This proof-of-concept indicates that MSC-derived membrane coatings, a technology already validated in other cancer and CNS settings, are now emerging in GBM research, bridging the translational gap.

Cell membrane-coated NPs collectively constitute a diverse and fast advancing platform that integrates the benefits of nanotechnology with the functional complexity of living cells. Their capacity to traverse intricate immunological and anatomical obstacles establishes them as a highly potential next-generation therapeutic approach for GBM, contingent upon the systematic resolution of existing challenges related to scale, standardization, and long-term safety.

### 5.7. Synthetic Mimics and Hybrid Systems (Engineered Vesicles, Exosome–Liposome Hybrids)

The engineering of hybrid delivery systems that combine the biological advantages of cell-derived vesicles with the tunability of synthetic nanomaterials has emerged as a leading frontier in GBM therapeutics. Unlike conventional EVs, these synthetic–biological hybrids allow programmable cargo loading, rational surface modification, and more precise control over biodistribution, release kinetics, and tumor tropism. As such, hybrid vesicular systems provide an adaptable platform capable of addressing several limitations of stand-alone biological or synthetic carriers. A key example of this approach is provided by Cao et al. (2023), who developed mesoporous polydopamine NP loaded with a small activating RNA (saRNA) designed to upregulate ALOX15 and induce ferroptosis in GBM cells [214]. These NPs were coated with macrophage membranes engineered to display Angiopep-2, significantly improving BBB penetration and reducing mononuclear phagocyte clearance. In orthotopic GBM models, this biomimetic system achieved high tumor accumulation, disrupted mitochondrial integrity, and triggered ferroptotic cell death, illustrating how hybrid platforms can integrate membrane-derived tropism with mechanism-specific cytotoxicity.

Li et al. (2023) conducted a representative investigation in which they engineered mesoporous silica NP coated with macrophage membranes and encapsulated with an anti-NF-κB peptide [225]. Post-radiotherapy, these hybrid particles inhibited the proneural-to-mesenchymal transition of GSCs, a significant contributor to therapeutic resistance, and improved survival in mice with GBM. This study emphasizes the engineering of membrane-coated hybrids for enhanced localization and targeted regulation of tumor progression [225].

Collectively, these studies illustrate that hybrid vesicle–NP systems confer multiple therapeutic benefits, including enhanced intratumoral retention due to the inherent tropism of macrophage or stromal-cell membranes; facilitation of controlled release of drugs, RNA, or cytotoxic agents, such as ferroptosis inducers or pathway-specific inhibitors; modular design that allows for the combination of therapeutic cargo, targeting ligands (e.g., Angiopep-2, anti-NF-κB peptides), microenvironment-responsive triggers (e.g., hypoxia, pH), and immune-evasive biomimetic surfaces; and improved BBB penetration attributed to the biological “passport” conferred by cell membranes.

Despite these promising attributes, hybrid systems face nontrivial challenges for clinical translation. These include GMP-compliant large-scale manufacturing, batch-to-batch reproducibility of membrane coating and cargo loading, incomplete understanding of biodistribution in humans, potential immunogenicity of membrane components, and uncertainties regarding long-term safety—such as off-target accumulation or unintended interactions with healthy neural tissue [231].

Alongside macrophage-based hybrid systems, progressively advanced platforms utilizing EVs produced from mesenchymal stem cells have emerged, attributed to their advantageous stability, payload capacity, and transport efficacy. For example, MSC-derived EV-lipid hybrids generated using microfluidic sonication have been utilized for mRNA administration in non-GBM malignancies [232], demonstrating the potential of integrating EV biocompatibility with synthetic lipid adaptability. A significant instance in the GBM domain is the development of MSC-derived spheroidal hybrids designed to express TRAIL and enclose nanocomposite pharmaceuticals. The spheroids facilitated the simultaneous delivery of TRAIL and mitoxantrone (MTX), leading to significant suppression of GBM proliferation both in vitro and in vivo, and exhibited enhanced targeting ability relative to genetically modified MSCs alone [233].

Beyond this example, however, the literature on engineered MSC-EV hybrids or vesicle-based mimetics specifically for GBM remains sparse, with most studies exploring these platforms in other cancer types. This gap underscores both the translational potential and the unmet opportunity for applying hybrid EV technologies in GBM therapy.

Synthetic mimics and hybrid vesicular systems signify a conceptual advancement from “cellular allies” to biomimetic technology allies, adept at integrating cellular intelligence with nanoscale programmability. Their ability for accurate, multi-modal, and BBB-permeable administration establishes them as one of the most promising emerging techniques for next-generation GBM therapies.

## 6. Overcoming Biological Barriers for Cell-Based Therapies

### 6.1. Strategies to Cross or Bypass the BBB

Despite the extensive interest in macrophage- and MSC-based delivery systems, crossing the BBB remains one of the least resolved challenges in GBM therapeutics. Many approaches rely on biological assumptions that are only partially supported by experimental evidence, and success in preclinical models has not consistently translated into human settings. Accordingly, BBB-oriented strategies must be evaluated critically and with realistic expectations.

**Figure 6:**
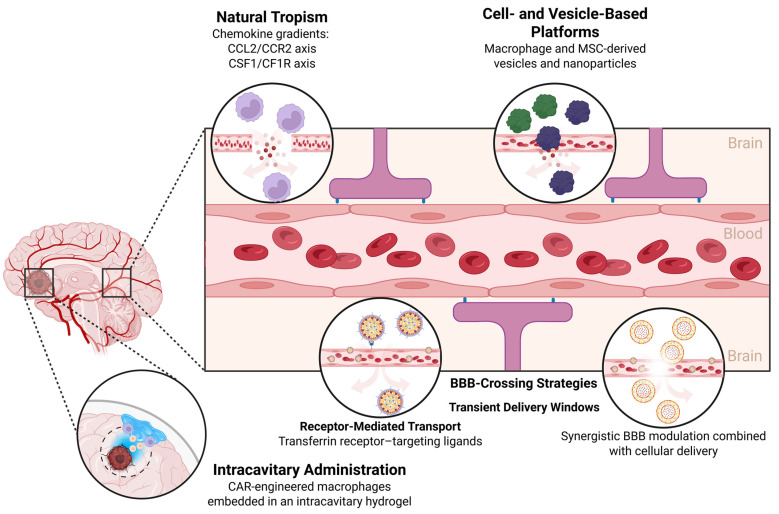
Strategies to overcome the BBB in GBM. This schematic summarizes complementary approaches that enable therapeutic access to the brain parenchyma in GBM. These include exploitation of the natural tropism of circulating monocytes and macrophages toward tumor-associated vascular disruption; the use of EVs or cell membrane-coated NPs that exploit transendothelial migration pathways; direct intracavitary administration using platforms capable of generating therapeutic cells in situ following surgical resection; the engineering of NPs or cellular carriers functionalized with ligands that promote receptor-mediated transport across the brain endothelium; and combinatorial strategies that leverage transient BBB perturbation to facilitate the targeted entry of cells or nanomaterials into the TME. Created in BioRender. Ibarra, L. (2026) https://BioRender.com/tetlisw (accessed on 12 December 2025).

First, although myeloid cells display a natural capacity to enter the CNS, their migration into GBM is neither uniform nor guaranteed. Chemokine axes such as CCL2/CCR2 or CSF1/CSF1R can recruit macrophages, but the degree of BBB disruption is spatially heterogeneous, and recruitment often reflects tumor-driven inflammatory cues rather than a reliable therapeutic route. Thus, while cell carriers can exploit these partially permeabilized regions, their penetration is largely constrained by the tumor’s own biology, which limits the predictability of this strategy. As schematically illustrated in Figure 6, immune-cell transmigration, vesicle passage, and NP transport predominantly occur in regions of pathological vascular permeability rather than through an intact BBB.

Second, vesicle- or membrane-coated NPs are often proposed as “hitchhikers” of leukocyte-like transmigration. However, direct evidence that these systems actively traverse an intact BBB remains weak. Most successful preclinical examples rely on models with significant vascular leakiness or post-irradiation damage, conditions that may not accurately represent the clinical BBB in non-enhancing or infiltrative GBM regions. Consequently, these platforms should be understood as exploiting pathological permeability rather than truly overcoming the physiological barrier. Third, intracavitary or loco-regional delivery avoids the BBB entirely and represents a more pragmatic solution. Approaches such as intracavitary hydrogels that generate or release engineered macrophages [171] bypass the barrier but introduce new challenges: limited diffusion in dense parenchyma, dependency on surgical access, and lack of control over spatial migration. While promising, these systems do not address infiltrative tumor cells distant from the resection cavity, which remain shielded by an intact BBB. Fourth, ligand-decorated cells or NP, targeting transferrin receptor, LRP1, or endothelial transcytosis pathways, offer mechanistic appeal, yet saturation, competitive binding, and receptor downregulation severely restrict transport capacity. Many such systems increase brain accumulation only marginally compared to unmodified carriers. Their utility in GBM must therefore be interpreted cautiously.

Finally, combining temporary BBB disruption (e.g., focused ultrasound, radiotherapy-induced permeability, PDT) with cell or NP delivery is conceptually attractive but raises concerns about safety, edema, neuroinflammation, and off-target leakage into healthy brain, particularly in a malignancy already associated with significant mass-effect and vascular fragility.

### 6.2. Navigating the Immunosuppressive TME

The immunosuppressive TME of GBM remains one of the major barriers to the success of cellular, immune, and NP-based therapies. Although numerous studies describe strategies to reprogram TAMs or enhance T-cell recruitment, the field has yet to overcome a series of persistent limitations that greatly constrain therapeutic efficacy [234,235]. Initially, GBM-associated myeloid populations demonstrate significant flexibility. Attempts to redirect TAMs towards proinflammatory states by CSF1R inhibition, TLR agonists, or pathway inhibitors (STAT3, PI3K) frequently result in inadequate or temporary reprogramming, as local cytokines and metabolic signals swiftly reinstate an immunosuppressive phenotype [236,237]. This underscores a critical issue: TAM regulation emerges within a TME that is physically and physiologically adapted to thwart immune activation, rendering sustained reprogramming challenging to accomplish. Secondly, the integration of cellular treatments with immune-checkpoint inhibitors (ICIs) has elicited much enthusiasm; yet preclinical data indicates that myeloid-driven dysfunction often constrains the efficacy of ICIs [238]. Although many studies indicate enhanced CD8^+^ infiltration following TAM management or gene therapy, these findings are predominantly based on preclinical models exhibiting elevated immunogenicity and frequently do not mimic the significant T-cell exclusion observed in real GBM. Consequently, the justification for combining TAM-targeted therapies with ICIs should be approached with caution, necessitating the identification of more definitive biomarkers to ascertain which individuals may derive benefit. Third, efforts to disrupt particular immunosuppressive pathways, such as PTEN-deficient LOX–OLFML3 signaling, demonstrate that localized alterations in chemokine networks can enhance T-cell infiltration [239]; however, these effects frequently rely on tumor-genotype-specific susceptibilities that may not be applicable to subset of GBMs. These findings highlight a significant limitation: myeloid-targeted therapies in GBM are improbable to be universally beneficial, and their translational efficacy relies on meticulous molecular classification.

In summary, whereas modified macrophages, MSCs, and other cell-based systems can influence the GBM TME, their efficacy is constrained by an environment that actively inhibits immune function. Addressing this obstacle necessitates strategies that concurrently: (i) disrupt the metabolic and cytokine pathways that maintain myeloid suppression, (ii) establish prolonged periods of T-cell permissiveness, and (iii) integrate patient-specific immunogenomic characteristics instead of depending on generalized models of tumor-associated macrophage behavior.

### 6.3. Enhancing Intratumoral Penetration and Retention

Achieving adequate distribution and sustained retention of therapeutic cells, vesicles, or NPs within GBM remains an unresolved challenge. Although hydrogels [240,241], biomimetic coatings [242], and convection-enhanced delivery (CED) [243] have expanded the repertoire of available strategies, they operate within a microenvironment whose physical and biological constraints are considerably more complex than those reproduced in standard preclinical models. The abnormal and mechanically rigid ECM in GBM restricts molecular and cellular diffusion, confining most therapies to limited regions of the tumor mass and preventing effective access to infiltrative margins. Materials such as hydrogels and scaffolds can prolong local persistence, yet improved retention rarely translates into comprehensive coverage, particularly for therapies that must reach cells migrating along white-matter tracts or perivascular routes. Matrix-responsive systems, designed to release their payload in response to enzymatic or pH cues, offer more spatially tailored delivery, but their behavior is strongly influenced by microregional variability. The biochemical landscape differs sharply between tumor core, invasive front, and adjacent brain tissue, raising concerns that release kinetics observed in murine models may not reflect performance in human GBM.

Similarly, while CED can enhance volumetric dispersion, its clinical implementation is hampered by backflow, variability in catheter placement, and reduced efficacy in areas of high interstitial pressure. The need for specialized expertise and the difficulty of performing repeated infusions further limit its practical utility for treatments that require sustained exposure.

Biomimetic carriers, including macrophage membrane-coated NPs and EV-loaded hydrogels, have demonstrated superior intratumoral accumulation in rodent models. However, these outcomes rely heavily on murine stromal architecture and immune composition, which differ substantially from human GBM [244]. Whether similar patterns of penetration and retention occur in patients remains largely speculative.

Improving intratumoral distribution will therefore require delivery platforms capable of adapting to the spatial heterogeneity of GBM, technologies that account for anatomical and biomechanical constraints, and systems that can engage not only the tumor bulk but also the invasive extensions that lie beyond the surgical cavity. Current approaches address these needs only partially, underscoring the importance of integrated strategies for meaningful clinical translation. Ensuring that therapeutic macrophages, stromal cells, or vesicle-based carriers penetrate GBM tissue and remain long enough to exert meaningful effects is a major challenge, given the dense ECM, elevated interstitial pressure, and heterogeneous vascular architecture.

### 6.4. Targeting GBM Heterogeneity and Invasive Cells

The extraordinary heterogeneity of GBM—encompassing genetic, epigenetic, metabolic, spatial, and immunological dimensions—continues to undermine the durability of current and emerging therapies. While many cell-based and nanotechnology platforms achieve substantial effects against bulk tumor populations, they frequently fail to engage the invasive and treatment-resistant subpopulations that drive recurrence, such as GSCs and migratory progenitors.

Even advanced delivery systems that accumulate efficiently in the tumor core often show inconsistent penetration into peripheral infiltrative regions. These areas tend to preserve elements of an intact BBB, exhibit reduced inflammatory signaling, and maintain distinct ECM characteristics, all of which collectively impair the access of engineered carriers that otherwise display promising tropism.

Beyond immune and stromal constraints, intrinsic tumor cell phenotypes also pose significant barriers to the success of cell-based therapies in GBM. Among these, polyploid giant cancer cells (PGCCs) represent a distinct, stress-induced subpopulation characterized by large cell size, polyploidy, and stem-like properties that contribute to tumor progression and therapeutic resistance. PGCCs can arise through mechanisms such as endoreplication, cell fusion, or mitotic failure in response to cytotoxic stressors including hypoxia and therapy-induced damage [245]. These cells have been implicated in promoting tumor heterogeneity, generating progeny with enhanced survival and invasive capabilities, and sustaining tumor repopulation after treatment, thereby undermining durable responses to conventional and emerging therapies [246]. Importantly, PGCCs actively modulate the TME, including through the secretion of factors that influence surrounding cellular populations and may reinforce immunosuppressive circuits that impede effective immune engagement. Consequently, even when macrophage reprogramming or immune recruitment are partially achieved, the persistence of PGCCs and their progeny may enable tumor regrowth and limit the long-term efficacy of cell-based strategies. Recognizing PGCCs as a relevant biological barrier underscores the need to develop therapeutic approaches that not only address immunosuppressive myeloid networks but also target resilient tumor cell states that contribute to resistance and relapse.

Therapeutic strategies directed toward specific GSC phenotypes, such as those aimed at suppressing the proneural-to-mesenchymal transition or sensitizing stem-like niches to radiotherapy; demonstrate encouraging preclinical activity [225]. Yet their success hinges on the prevailing transcriptional state of the tumor, which is dynamic and subject to rapid adaptation under therapeutic pressure. This plasticity greatly limits the durability of interventions focused on single pathways or phenotypes.

Emerging multifunctional platforms, including ferroptosis-inducing NPs, localized hydrogel–liposome hybrids [247], and glutathione-responsive prodrug systems [248], attempt to exploit vulnerabilities enriched in resistant subpopulations. Despite their sophistication, these technologies often act within restricted compartments of the tumor and rarely achieve widespread coverage across diverse cellular niches.

Moreover, numerous delivery systems are assessed using models that poorly represent the invasive characteristics of human GBM. Standard orthotopic xenografts exclude essential stromal and immunological components and inadequately mimic the diffuse infiltration patterns observed in patients, prompting concerns that preclinical efficacy may exaggerate the ability of these platforms to eliminate clinically significant invasive cells.

Effectively addressing GBM heterogeneity will require therapeutic architectures that are modular, adaptable, and capable of engaging spatially and phenotypically distinct tumor regions. Solutions will likely integrate biomimetic carriers with complementary mechanistic targets and localized delivery systems designed to control both the tumor core and its infiltrative extensions. Such approaches offer the best prospect for overcoming the layered and evolving resistance mechanisms that characterize this disease.

## 7. Preclinical Models and Evaluation Metrics

### 7.1. In Vitro Models: 2D Cultures, Spheroids, and Organoids (3D)

Two-dimensional (2D) cultures are extensively utilized for examining interactions between stromal cells, such as MSCs or immune cells such as TAMs, and GBM cells due to their simplicity, cost-effectiveness, and stringent experimental control [249]. These platforms are particularly useful for interrogating the direct effects of MSC-derived secretome components, viral vectors, anticancer agents, or therapeutic microRNAs on tumor proliferation, migration, and survival. Many studies employ direct 2D co-cultures [195,208,209,250], whereas others use transwell systems to isolate paracrine interactions without direct physical contact [178]. Transwell inserts, typically with pore sizes around 0.4 μm, maintain spatial separation between MSCs and GBM cells while allowing soluble mediators to diffuse—an approach that helps dissect paracrine signaling pathways.

Despite these advantages, 2D systems lack the architectural and microenvironmental complexity of GBM tissue, including ECM structure, oxygen gradients, and cellular heterogeneity. These limitations have driven increasing adoption of three-dimensional models such as spheroids, organoids, and organotypic brain slices, which better recapitulate spatial organization, hypoxic niches, and the dynamic crosstalk of the TME [249,251,252].

Organoids and organotypic slices are particularly valuable for evaluating MSC or TAM infiltration, therapeutic efficacy of cell-delivered agents, and modulation of tumor niches. For example, Kurogi et al. (2019) employed organotypic brain slices to test MSCs engineered to co-express hsa-miR-145-5p and hsa-miR-31-5p, demonstrating reduced glioma invasiveness, increased apoptosis, and cell-cycle arrest [250]. These findings highlight the superior predictive power of 3D systems for assessing stroma–tumor interactions.

### 7.2. In Vivo Models: Orthotopic Xenografts, Syngeneic Models, and Genetically Engineered Mice

To synthesize emerging evidence, Table 5 summarizes recent in vivo studies, detailing MSC or EV dosage, delivery routes, tumor models, and therapeutic outcomes. A consistent pattern emerges across these reports: MSCs used as carriers of therapeutic payloads generally produce measurable antitumor effects, including reduced tumor growth and prolonged survival, regardless of the exact number of administered cells. These findings reflect the intrinsic tumor-homing capacity of MSCs and their ability to act as localized delivery vehicles.

Spatial proximity, however, strongly influences therapeutic performance. Intratumoral and peritumoral injections typically yield higher MSC retention and more robust biological activity, whereas systemic routes such as intravenous or intra-arterial delivery generate variable responses that depend heavily on tumor tropism and microenvironmental cues. Notably, several studies report minimal systemic toxicity, further supporting the clinical feasibility of MSC-based strategies.

Biodistribution studies reinforce the importance of delivery route and tumor state. Mao et al. (2023) used MSCs co-expressing CXCL10, Nrf2, and ferritin (FTH), enabling iron-sensitive MRI tracking [179]. Their data demonstrated preferential MSC retention and sustained functional activity following peritumoral administration. Yudintceva et al. used SPIONs and DiR labeling to show that MSC migration from systemic circulation depends strongly on tumor-associated inflammation and angiogenesis, suggesting increased homing efficiency in more advanced tumors [104]. Yueh et al. (2025) likewise showed that carotid-artery injection enhances intracranial delivery and tumor-directed localization [184].

These findings collectively demonstrate that MSC biodistribution is a dynamic, context-dependent process influenced by the injection method and the condition of the TME. Local distribution regularly improves retention and therapeutic durability, while systemic administration may necessitate tailored enhancements, such as enhanced migratory capacity or molecular targeting, to attain similar efficacy.

In conclusion, existing evidence supports localized approaches for MSC-based treatments in GBM. Systemic approaches have potential but will likely necessitate advanced engineering or preconditioning techniques to surmount the significant obstacles presented by GBM biology and the BBB.

## 8. Clinical Evidence and Translational Progress of Macrophage- and MSC-Based Therapies in GBM

Clinical evaluation of macrophage- and MSC-based therapies in GBM is still at an early stage, yet recent studies provide important proof-of-concept evidence supporting their feasibility in humans. A major milestone was the first-in-human trial of CAR macrophages, CT-0508, targeting HER2. In this phase I study in HER2-positive solid tumors (NCT04660929), Klichinsky et al. showed that CAR-M can be manufactured, infused safely, infiltrate tumors, and remodel the microenvironment toward a proinflammatory state, all without significant toxicities. Although GBM was not included, the trial represents the first clinical validation that genetically engineered macrophages can function as therapeutic agents in humans.

In GBM specifically, the most advanced clinical experience involves MSCs used as vehicles for oncolytic viruses. The DNX-2401 (Delta-24-RGD) adenovirus, initially delivered intratumorally in recurrent GBM, demonstrated safety and durable responses in subsets of patients (NCT03896568). Building on these findings, bone-marrow–derived MSCs have been loaded ex vivo with DNX-2401 and administered intra-arterially to enhance viral dispersion within the tumor. Preliminary results from the ongoing phase I study show favorable tolerability and successful intratumoral release of the virus.

Overall, current clinical efforts emphasize early endpoints such as safety, biodistribution, and immune activation, with a strong focus on local or regional administration to bypass the BBB. These studies are laying the groundwork for next-generation combination regimens that may integrate macrophage engineering, MSC-mediated delivery, oncolytic viruses, and systemic immunomodulators.

## 9. Translational Readiness: Efficacy Endpoints, Biomarkers, and Lessons from Clinical Experience

Advancing macrophage- and MSC-based therapies from preclinical promise to clinical applicability in GBM requires far more than demonstrating tumor suppression in animal models. As the evidence reviewed here indicates, cargo-loaded MSCs and engineered macrophages consistently exhibit tumor tropism and therapeutic activity in controlled laboratory settings. However, the primary translational obstacles persist: quantifiable biodistribution, real-time monitoring of cellular fate, functional verification of therapeutic payload activation, and comprehensive safety assessment within the highly heterogeneous GBM microenvironment.

Preclinical imaging strategies, including iron-sensitive MRI, SPION labeling, DiR fluorescence, or overexpression of ferritin reporters, have enabled important insights into MSC or macrophage trafficking. Nonetheless, these methodologies exhibit inherent limitations: signal decay, label dilution with cell division, off-target uptake, artifacts from dead cells, and limited sensitivity near detection thresholds. None satisfy the regulatory standards for quantitative, reproducible, human-grade cell tracking. This gap underscores the need for clinically validated imaging platforms such as PET reporter genes, magnetic particle imaging (MPI), or hybrid multimodal systems capable of measuring absolute cell numbers, longitudinal persistence, route-dependent biodistribution, and payload activation in vivo. Without such tools, dose optimization, safety assessment, and mechanistic interpretation in first-in-human trials will remain incomplete.

Regulatory considerations add further complexity. Therapies based on MSCs, EVs, or engineered macrophages must demonstrate cellular identity, genomic stability, absence of tumorigenic potential, and predictable functional behavior, despite the intrinsic heterogeneity of MSCs and the context-dependent plasticity of myeloid cells. Preclinical systems often overestimate therapeutic consistency because local administration (intratumoral or peritumoral) bypasses barriers, such as the BBB and peripheral clearance routes, that dominate human physiology. Moreover, the field still lacks validated biomarkers capable of predicting which tumors will most effectively recruit therapeutic cells, sustain viral replication (for oncolytic virus–MSC strategies), or support macrophage polarization toward antitumoral states.

Clinical experience with related platforms has yielded equally important lessons. Virotherapy trials, including CAN-3110 and DNX-2401, have shown that engagement of innate and myeloid immunity, particularly M1-like polarization and interferon signaling, correlates with improved survival [255,256]. These findings highlight a principle that is directly relevant to cell-based delivery systems: the therapeutic efficacy of engineered cells depends as much on the immunological state of the TME as on the properties of the therapy itself. In the DNX-2401 + pembrolizumab trial, intratumoral injection was feasible and safe, but durable clinical responses occurred only in tumors capable of sustaining viral propagation and mounting a robust secondary immune reaction, criteria that vary markedly between patients. These outcomes emphasize the need for stratification biomarkers, potentially including baseline macrophage signatures, interferon response modules, or spatial features from multiplex imaging, to identify patient subsets most likely to benefit from cell- or EV-based interventions.

Similarly, the early-phase clinical testing of CAR macrophages (CT-0508) has validated the feasibility of manufacturing, genetic engineering, and safely infusing myeloid therapies in humans. However, translation into GBM remains constrained by uncertainties in BBB transit, in situ persistence, and risks of neuroinflammation. For macrophage-, MSC-, or EV-based strategies, the next generation of clinical trials will likely require integrated biomarker frameworks capable of monitoring: therapeutic-cell localization, dynamic reprogramming of TAM/microglia, emergence of inflammatory or off-target toxicities, and tumoral resistance trajectories.

The insights from virotherapy and first myeloid engineering studies emphasize that effective translation in GBM necessitates the convergence of modalities rather than their isolation. Recent work increasingly indicates multimodal regimens that integrate myeloid reprogramming (e.g., CSF1R blocking, CD47/SIRPα suppression), checkpoint inhibitors, cell- or EV-based delivery methods, and molecular biomarkers for patient selection and adaptive dosing.

The future of macrophage- and MSC-derived therapies depends on addressing the measurement issue: without precise, clinically applicable quantitative biomarkers for biodistribution, persistence, and biological activity, therapeutic effects cannot be accurately interpreted, optimized, or expanded. The shift from preclinical observation to clinical reliability will rely on the integration of biological innovation with accurate, validated instruments for monitoring the mechanisms and locations of action of these live treatments within the intricate ecology of human GBM.

## 10. Biological Safety Risks and Challenges for Cell-Based and EV-Based Therapies

Macrophage- and MSC-based therapies, including cell-free strategies using EVs, offer promising alternatives to conventional chemo-radiotherapy for GBM, yet they introduce a distinct spectrum of biological, immunological, and translational risks. Aggregated clinical data from EV-based trials across indications suggest a low incidence of serious adverse events; however, reporting remains heterogeneous, and follow-up periods are short [257]. In GBM-directed studies, only a limited fraction of preclinical investigations systematically address safety, and long-term toxicities, neuroinflammatory sequelae, and immune reprogramming effects remain insufficiently characterized [258]. Likewise, early-phase clinical trials of cell-based therapies are typically underpowered to detect delayed or rare adverse events, focusing primarily on feasibility and acute safety.

A central biological concern is the context-dependent behavior of therapeutic cells within the GBM microenvironment. MSCs and macrophages possess potent immunomodulatory and trophic functions that can be either therapeutically beneficial or deleterious depending on microenvironmental cues. While engineered MSCs have been shown to deliver cytotoxic genes, immune-stimulatory cytokines, or pro-apoptotic ligands, unmodified or inadequately controlled MSCs may secrete factors such as VEGF, TGF-β, or IL-6 that promote angiogenesis, invasion, EMT, and immunosuppression. Consequently, MSCs have been reported to either suppress or enhance glioma growth depending on cell source, dose, delivery route, and tumor context, underscoring the unpredictability of their net biological effect. Similar plasticity applies to macrophage-based strategies, where therapeutic reprogramming may be overridden by the profoundly immunosuppressive GBM TME, favoring a tumor-supportive M2/TAM-like phenotype.

Immune-mediated toxicities represent a particularly critical risk in the GBM setting. Adoptive immune-cell therapies, including macrophage-based platforms and genetically engineered cells, carry the potential for cytokine release syndrome (CRS) and immune effector cell–associated neurotoxicity syndrome (ICANS) [259]. In GBM, these risks are amplified by the frequent use of intracranial or locoregional delivery routes, such as intratumoral, intracavitary, or intraventricular administration, designed to bypass the BBB. While effective for improving local drug exposure, these approaches can paradoxically intensify neuroinflammation within an already vulnerable compartment. In this context, CRS may manifest not only as systemic inflammation but as intracranial hypertension, severe headache, nausea, or altered consciousness. ICANS-like neurotoxicity in GBM patients could present as exacerbated peritumoral edema, obtundation, seizures, or catastrophic cerebral edema leading to herniation. Managing these complications is particularly challenging in patients with compromised brain compliance, mass effect, and pre-existing neurological deficits, making neurotoxicity a potentially dose-limiting and life-threatening adverse event.

Immunogenicity and immune compatibility further complicate translation. Allogeneic MSCs or engineered macrophages may be recognized and cleared by the host immune system, triggering inflammatory reactions or loss of therapeutic efficacy [260]. Conversely, both MSCs and macrophages possess intrinsic immunosuppressive properties that may dampen endogenous antitumor immunity or interfere with immune checkpoint therapies. Although EVs are often described as minimally immunogenic due to the absence of nuclei and major histocompatibility complexes, they retain parent-cell proteins, lipids, and RNAs capable of modulating immune responses. While acute toxicity following EV administration appears limited in animal models, the safety of high-dose or repeated EV exposure remains poorly defined.

Off-target biodistribution and delivery inefficiency constitute additional biological hazards. Following intravenous infusion, a substantial proportion of administered MSCs become sequestered in pulmonary capillaries, with only a small fraction reaching the brain [261]. Cell aggregation may lead to microvascular obstruction and thromboembolic events, while engineered cells or EVs that localize to non-target organs may exert unintended biological effects. In the CNS, any form of intracranial delivery carries inherent risks, including focal inflammation, seizures, and edema, further narrowing the therapeutic window. These distribution constraints highlight persistent challenges in achieving precise, safe, and effective targeting of GBM lesions.

Manufacturing-related risks further intersect with biological safety. MSC products are intrinsically heterogeneous, with donor variability, passage number, and culture conditions profoundly influencing phenotype, secretome composition, and EV cargo. Prolonged expansion may introduce genetic or epigenetic instability, necessitating rigorous genomic surveillance. Viral vector-based engineering adds additional concerns related to insertional mutagenesis, vector persistence, and regulatory complexity. For EV-based products, scalable GMP-compliant manufacturing, cargo standardization, and elimination of co-isolated contaminants remain unresolved challenges with direct implications for safety and reproducibility.

In summary, while macrophage-, MSC-, and EV-based therapies represent innovative and potentially transformative approaches for GBM, they face a complex safety landscape encompassing immune-mediated neurotoxicity, protumorigenic risks, off-target effects, and manufacturing variability. Meaningful clinical translation will require integrated risk-mitigation strategies, including conservative dosing, inducible safety switches, local versus systemic delivery optimization, quantitative biodistribution imaging, and biomarker-driven patient selection, to ensure that therapeutic benefit outweighs biological risk in this uniquely fragile clinical context.

## 11. Scalable Manufacturing and GMP Considerations for Cellular and EV-Based Products

For cell-based therapeutics, the cells themselves, or their secreted products, constitute the final medicinal product. Efficient and scalable cell culture is therefore a critical requirement for clinical translation. As outlined in Section 5, a wide range of genetic engineering strategies can be employed to achieve overexpression of therapeutic payloads. One commonly used approach is second-generation lentiviral transduction, which has demonstrated favorable performance in expanding MSC cultures in suspension using microcarriers within bioreactors. Enhanced cellular adhesion has even been reported when microcarriers are coated with plastic, fibronectin, or collagen [262].

Despite these advantages, the rapid proliferation of lentivirally transduced MSCs is counterbalanced by significant translational limitations. Numerous authors now recognize second-generation lentiviral transduction as a major bottleneck for clinical scalability. Lentiviral vectors introduce several constraints in the GMP setting, including difficult purification, high production costs, random genomic integration, the requirement for biosafety level 3 (BSL-3) facilities, and the need to establish batch-specific release criteria. Collectively, these barriers impede large-scale implementation. Moreover, in MSCs specifically, lentiviral engineering can impair proliferation and promote cellular senescence [208].

In response to these challenges, alternative nonviral approaches have been proposed. One promising strategy involves transfection using the cationic polymer PEI, supplemented with fusogenic lipids and β-tubulin deacetylase inhibitors, to express yeast cytosine deaminase/uracil phosphoribosyltransferase/green fluorescent protein (CD::UPRT::GFP). This method has been successfully scaled to T175 flasks and to microcarrier-based bioreactors, achieving transfection efficiencies above 90% without compromising MSC migration or homing properties. Importantly, CD::UPRT::GFP-expressing MSCs exhibited robust antitumor activity against TMZ-resistant GBM in both in vitro and in vivo models. These results indicate that PEI-mediated transfection represents a scalable and clinically viable method for generating large quantities of engineered MSCs with high therapeutic potential [208].

Beyond the choice of genetic engineering method, the culture substrate on which MSCs adhere and expand is fundamental. Stirred-tank bioreactors combined with microcarriers enable large-scale MSC production [263]. In addition to plastic-, fibronectin-, or collagen-coated microcarriers [262], serum-free microcarriers specifically designed for MSC culture have been developed. These include hyaluronic acid-coated carriers, 100–200 μm in diameter and concave in structure, which support robust MSC proliferation [263]. Similarly, MSC-derived EVs have been successfully isolated from stirred bioreactors seeded with microcarriers, yielding up to 1.7 × 10^11^ EVs per batch under serum-free conditions [264]. EV secretion can be further enhanced when microcarriers create localized hypoxic conditions for proliferating MSCs [265].

Taken together, advances in genetic engineering strategies and scalable adhesion substrates now make it feasible to generate large quantities of therapeutic MSCs and EVs suitable for clinical-grade manufacturing. However, protocol harmonization remains a major unmet need. Few studies, aside from Tu et al. (2020) [208], have demonstrated scalable engineering of MSCs capable of producing antitumor EVs at clinically relevant yields. Continued refinement of GMP-compliant workflows is therefore essential to support the translation of MSC- and EV-based therapies into the clinical arena for GBM.

## 12. Conclusions and Future Outlook

GBM remains one of the most treatment-refractory human malignancies, shaped by profound cellular heterogeneity, diffuse invasion, and a strongly immunosuppressive microenvironment. The limitations of conventional therapeutics—restricted BBB penetration, insufficient intratumoral distribution, and rapid emergence of resistance—have driven the search for alternative strategies capable of navigating the unique biology of GBM. Macrophages and MSCs have emerged as compelling candidates in this landscape, distinguished by their intrinsic tumor tropism, ability to remodel the microenvironment, and compatibility with advanced engineering approaches.

The evidence synthesized in this review demonstrates that cell-based systems—whether used directly as therapeutic agents, engineered as genetically modified effectors, or harnessed as carriers for NPs, biologics, or oncolytic viruses—have achieved significant mechanistic advances across preclinical models. Macrophage-derived and MSC-derived vesicles, membrane-coated NPs, and hybrid biomimetic systems illustrate how cellular biology can be coupled with materials science to overcome major barriers such as the BBB, stromal resistance, and immune suppression. Yet the translation of these platforms remains hampered by critical uncertainties: the complexity of human GBM architecture relative to animal models, incomplete understanding of long-term persistence and fate of administered cells, and challenges in manufacturing, standardization, and safety assessment.

A central lesson emerging from related clinical experiences, particularly oncolytic virotherapy, immune-modulating strategies, and the first trials of CAR-macrophages, is that efficacy in GBM will require more than a single mechanistic intervention. Durable benefit will likely depend on combinatorial designs that coordinate myeloid remodeling, adaptive immune activation, targeted cytotoxicity, and spatially optimized delivery. The development of quantitative biodistribution imaging, standardized potency assays, and early mechanistic biomarkers will be essential to guide first-in-human trials and to bridge the translational gap that has historically limited progress in GBM therapeutics.

Looking ahead, the field is poised to transition from proof-of-concept studies toward rationally engineered, clinically informed platforms. Next-generation strategies will likely incorporate modular cell engineering, programmable release systems, microenvironment-responsive biomaterials, and hybrid biological–synthetic constructs capable of adapting to the spatial and temporal heterogeneity of GBM. At the same time, advances in patient-derived organoids, spatial transcriptomics, and immunogenomics will provide the resolution required to align therapeutic design with the true complexity of human disease.

In summary, macrophages and MSCs represent versatile and biologically grounded allies against GBM, but their full therapeutic potential will be realized only through integrative approaches that combine engineering innovation, rigorous translational frameworks, and mechanistic precision. The path forward is challenging, yet the convergence of cellular immunotherapy, nanotechnology, and biomaterials offers a realistic opportunity to redefine treatment paradigms for a cancer that has long resisted therapeutic progress.

## Figures and Tables

**Table 2:** GBM molecular/histological subtypes, TAM–MSC milieu, and implications for cell-based therapies.

GBM Subtype/Grade (WHO/Molecular)	Histologic/Architectural Notes	Key Tumor-Intrinsic Features	TAM/Microglia Features	MSC/Stromal Features	Biomarkers for Patient Stratification	Implications for Cell-Based Therapies	Reference
Mesenchymal GBM (MES-GBM)/IDH-wt, WHO 4	Extensive necrosis; hypoxia; marked BBB disruption; microvascular proliferation.	NF1 loss; inflammatory and EMT-like transcriptional programs.	Highest immune and matrix infiltration; strong association with brain-resident microglia and monocyte-derived TAMs; TAM programs linked to MES pathogenesis and RT-induced infiltration; OSM-driven TAM → MES-like transition.	Abundant GA-MSCs; high CCL2/CSF1/IL-10 signaling. GA-MSC–glioma hybrids promote CSF1-dependent recruitment and M2 polarization.	NF1 status; TAM density (IHC, scRNA-seq); CSF1/CCL2 expression; contrast-enhancing MRI	Favorable for macrophage/MSC-based delivery but requires macrophage reprogramming or combination with immunomodulation. Enriched targets for TAM-reprogramming/depletion, anti-OSM/STAT3, and strategies that disrupt GA-MSC–TAM axes.	[77,78,151,152,153]
Classical GBM (IDH-wt)	Prominent vascular proliferation; heterogeneous BBB permeability	EGFR amplification; RTK-driven proliferation	Moderate TAM infiltration; mixed microglia/BMDM populations; Lower immune cell content than MES; less permissive to immune infiltration.	ECM/stroma less pronounced than MES, but remodeled in high-risk groups	EGFR amplification; perfusion MRI; PD-L1 expression; myeloid markers	May benefit from EGFR-targeted plus T-cell-based approaches; microenvironmental barriers moderate, Suitable for engineered macrophages or ligand-guided cell/EV delivery combined with cytotoxic or immune therapies.	[153,154,155]
Proneural GBM/IDH-mut astrocytoma WHO 4	Less necrosis; partially intact BBB; diffuse infiltration	Often in younger patients; may lack florid necrosis early. PDGFRA alterations; IDH mutations (subset); stem-like feature.	Lower myeloid burden; predominance of microglia-like TAMs. Lowest immunogenicity; reduced TAM/immune infiltration vs. MES and neural.	Less mesenchymal/ECM remodeling; stem-like programs and developmental hierarchies dominate.	IDH mutation; PDGFRA status; low contrast enhancement; microglia markers	Poor responders to aggressive chemoradiation; cellular therapies may require strategies overcoming “cold” TME and targeting stem-like compartment. May require intraparenchymal or intracavitary delivery and targeting of invasive tumor cells.	[151,153,156,157]
“Molecular GBM”/IDH-wt grade 2/3	Histology may appear lower grade; radiologic–molecular discordance.	Chromosomal instability with +7/−10 signature; TERT promoter mutations (C228T/C250T); EGFR amplification; diffuse infiltrative growth pattern	Immune/stromal composition variably resembles IDH-wt GBM; requires profiling.	As above, not histologically obvious, risk of under-treating TME.	IDH wild-type status; EGFR amplification (±EGFRvIII); TERT promoter mutation; MGMT promoter methylation; PTEN loss or PI3K–AKT pathway activation.	Molecular diagnosis should trigger full TME characterization and inclusion in trials of GBM-directed cellular therapies despite low-grade histology.	[151,152]
Non-enhancing/infiltrative regions	Intact BBB; diffuse parenchymal invasion	Slow-cycling, therapy-resistant tumor cells	Sparse immune infiltration; limited TAM.	Limited MSC density.	Non-enhancing MRI; molecular residual disease markers	Delivery strategies must bypass BBB (local delivery, EVs with targeting ligands)	[152,158]

Footnotes: WHO classification. Tumor entities and grades are defined according to the WHO Classification of Tumors of the Central Nervous System (5th edition). The term “molecular GBM” refers to IDH-wildtype diffuse astrocytic tumors of WHO grade 2 or 3 that fulfill molecular criteria for glioblastoma, including chromosome +7/−10, TERT promoter mutation, or EGFR amplification, irrespective of histological grade. Immune infiltration descriptors. Qualitative terms such as “high,” “moderate,” or “low” immune or stromal infiltration reflect consensus findings from immunohistochemistry, bulk RNA sequencing, and single-cell RNA sequencing studies rather than absolute quantitative thresholds. Biomarkers for stratification. Biomarkers listed include molecular (e.g., IDH status, EGFR amplification, TERT promoter mutation, MGMT promoter methylation), cellular (e.g., TAM density, MSC signatures), and imaging-based features (e.g., contrast enhancement, perfusion MRI). These markers are intended to support patient stratification, not as stand-alone predictive biomarkers. Therapeutic implications. Implications for cell-based therapies represent preclinical and translational interpretations derived from integrated molecular, histological, and microenvironmental data. They do not imply current clinical efficacy and should be considered hypothesis-generating. Infiltrative regions. Non-enhancing or infiltrative tumor regions are included as a functional compartment rather than a molecular subtype, as they represent a major reservoir of therapy-resistant GBM cells with distinct microenvironmental constraints.

**Table 3:** MSC-derived EVs and MSC-based delivery platforms evaluated in preclinical GBM models.

MSC/EV Source	Cargo/Modification	Experimental Model	Main Biological Effects	Key Outcomes	Reference
Wharton’s Jelly MSCs	miR-124	U87MG (in vitro, in vivo)	Cell cycle arrest, apoptosis induction	Reduced tumor growth, increased TMZ sensitivity	[178]
Adipose MSCs	miR-4731-5p	U-251, and U-87 cell lines co-cultures with MSCs	tumor-suppressor miRNA.	cell cycle arrest, reduce proliferation, and apoptosis in the GBM cell line	[180]
Umbilical cord MSCs and EV-derived MSCs	miR-124 and PD-1	GL261 and GBM8401 cells co-cultures with EVs (in vitro) and orthotopic GL261 model (in vivo)	Tumor suppressor miRNA and immune inhibitory regulator	Alterations in the TME were observed. Administration of exosomes and genetically modified MSCs reduced tumor volume and significantly prolonged animal survival. Importantly, the MSCs did not exhibit tumorigenic potential.	[184]
Murine MSCs	recombinant lentivirus encoding Cxcl10, Nrf2 (an anti-apoptosis gene), and a ferritin heavy chain (Fth)	orthotopic GL261 and CT2A GBMs (in vivo)	Enhanced CXCL10 secretion, Nrf2 expression, and MRI visibility.	Changes in the TME, increased infiltration of T lymphocytes, reduced tumor cell proliferation, and improved survival of treated animals.	[179]
Murine bone marrow-derived MSCs	IL-12 and nCD47-SLAMF7	CD8^+^ T cells and macrophages (in vitro) and GL261-luc orthotopic model (in vivo)	Secretion of IL-12 (anti-tumor immune cytokine) and nCD47-SLAMF7 fusion protein (regulates T-cell activity and macrophage phagocytosis).	Reduced tumor cell proliferation and improved animal survival, accompanied by alterations in the TME. Notably, the implanted MSCs underwent cell death within 8 weeks after implantation, indicating no evidence of tumorigenic potential.	[183]
Human adipose MSCs	yeast cytosine deaminase::uracil phosphoribosyl-transfease::green fluorescent protein (CD::UPRT::GFP)	Cocultures of AD-MSC with GBM cell lines U-251MG- and U-87MG-resistant cell lines to TMZ (in vitro) and U-251MG resistant to TMZ heterotopic mice model (in vivo).	cell-directed enzyme prodrug therapy (UPRT), cytosine deaminase/5-fluorocytosine (CD/5FC) prodrug system where CD converts a non-toxic prodrug (5-FC) into an anticancer drug 5-fluorouracil (5FU)	A significant reduction in tumor size and enhanced sensitivity to 5-FU were observed in vitro, irrespective of TMZ sensitivity.	[208]
Murine MSCs	yeast cytosine deaminase (CDA) and IL18-FC	Cocultures with GL261 (in vitro) and orthotopic GL261 model (in vivo)	in the presence of 5-FC, induced cell cycle arrest and apoptosis.	suppressed cancer progression, delayed tumor recurrence, and improved survival.	[185]
Murine adipose MSCs	IFNB y scFu-PD1	CT2A and GL261 cell lines (in vitro) and orthotopic mouse models (in vivo)	induces apoptosis in GBM tumor cells and upregulates PD-L1 expression.	enhances T-cell activation and T-cell-mediated tumor cell killing, resulting in reduced tumor growth and improved survival in mouse GBM tumors.	[209]
Murine bone marrow-derived MSCs	miR-133b	EV exposure to U87 cells (in vitro) and orthotopic U87 mouse model (in vivo)	EVs suppressed proliferation, invasion, and migration by inhibiting EZH2 and the Wnt/β-catenin signaling pathway.	tumor-suppressive effects of MSC-derived exosomal miR-133b on glioma development.	[210]
Human bone marro MSCs-derived EVs	miR-142-3p	EV exposure to SW1088 cells or A172 cells	EVs significantly suppressed glioma cell migration and invasion while concomitantly promoting apoptotic cell death.	The isolated EVs suppressed cellular proliferation and increased apoptosis through inhibition of GFI1.	[211]

**Table 4:** Macrophage-Based and Biomimetic Delivery Strategies for GBM.

Vehicle/Payload	Dose/Amount Administered	Experimental Model	Route of Administration	Proposed Mechanism	Key Outcomes	Reported Limitations	Reference
Human or murine macrophages loaded with chimeric oncolytic adenovirus OAd5/F35[E2F1]		Orthotopic murine GBM xenograft (in vivo)	Systemic or intracranial	Trojan-horse mechanism enabling macrophage-mediated viral delivery to tumor sites	Enhanced intratumoral viral delivery, increased tumor cell death, and prolonged survival compared with free virus	Preclinical data only; long-term persistence and safety require validation.	[212]
Allogeneic macrophages loaded with ferritin–drug conjugates (macrophage–drug conjugates)		Orthotopic murine GBM model (in vivo)	Intravenous	Macrophage homing enables selective drug release within TME.	Reduced tumor burden and significantly prolonged survival compared with free drug	Animal models only; translational safety and scalability remain unclear	[198]
Macrophages loaded with albumin-bound paclitaxel (nab-paclitaxel)	5 × 10^1^ cells/mL in microfluidic assays	GBM-on-a-chip microvascular model (in vitro)	Microfluidic perfusion	Trans-endothelial migration and localized drug release	Inhibition of GBM cell proliferation and migration	In vitro validation only; no in vivo data	[213]
Macrophage membrane-coated NPs		Orthotopic murine glioma model	Intravenous	Biomimetic membrane camouflage improves tumor targeting and immune evasion	Enhanced tumor accumulation and therapeutic efficacy versus uncoated NPs	Not based on live cells; human immunological compatibility remains unknown	[214]
Macrophage-derived membrane or vesicle-coated NPs		Orthotopic murine glioma models and in vitro assays	Systemic	Chemokine-guided targeting with microenvironment-responsive release	Improved tumor targeting and antitumor efficacy	Heterogeneity of preparations; limited clinical translation	[215]

**Table 5:** MSC-Based Cellular Therapies in GBM: In Vivo Evidence.

Therapeutic Cargo	In Vivo Model	Dose (MSCs/EVs)	Route of Administration	Key Outcomes	Reference
IL-12/IL-7 co-expression	Orthotopic GBM	1 × 10^5^ MSCs	Intratumoral	Reduced tumor growth, increased survival, durable antitumor immune memory	[253]
CXCL10, Nrf2, FTH	Orthotopic GBM	2 × 10^6^ MSCs	Peritumoral	Reduced tumor growth and prolonged survival	[179]
Delta-24-RGD oncolytic virus	Orthotopic GBM	1 × 10^6^ MSCs	Carotid artery	Prolonged survival and durable tumor remissions	[196]
HSV-TK	Orthotopic GBM	5 × 10^4^ − 4 × 10^5^ MSCs	Contralateral & intratumoral	Increased survival without documented systemic toxicity	[254]
Ad5-Ki67/IL-15 co-expression	Orthotopic GBM	1 × 10^5^ MSCs	Striatum	Enhanced therapeutic efficacy of the administered oncolytic virus	[195]
HSV-TK	Orthotopic GBM	Not specified	Intravenous	Prolonged survival without systemic toxicity	[182]
TRAIL	Orthotopic GBM	2 × 10^5^ MSCs	Intracranial	Tumor growth inhibition through apoptosis induction	[201]
CD::UPRT::GFP	Subcutaneous glioma	5 × 10^6^ MSCs	Intratumoral	Reduced tumor growth and improved survival	[208]
IFNβ + scFv/PD-1	Primary GBM	1.5 − 4 × 10^5^ MSCs	Intracranial & intratumoral	Reduced tumor burden, prolonged survival, and TME immune reprogramming	[209]
miR-124/PD-1 co-expression	Orthotopic GBM	2 × 10^5^ MSCs/1 × 10^9^ EVs	Carotid artery	Inhibition of tumor growth, increased survival, no documented toxicity	[184]

## Data Availability

Not applicable.
